# Artificial intelligence of things for sustainable smart city brain and digital twin systems: Pioneering Environmental synergies between real-time management and predictive planning

**DOI:** 10.1016/j.ese.2025.100591

**Published:** 2025-06-28

**Authors:** Simon Elias Bibri, Jeffrey Huang

**Affiliations:** Institute of Computer and Communication Sciences (IINFCOM), School of Architecture, Civil and Environmental Engineering (ENAC), Media and Design Laboratory (LDM), Swiss Federal Institute of Technology Lausanne (EPFL), 1015, Lausanne, Switzerland

**Keywords:** Sustainable smart cities, Artificial intelligence of things, Urban brain, Urban digital twin, Cyber-physical systems of systems, Real-time operational management, Strategic predictive planning, Environmental sustainability

## Abstract

Rapid urbanization, alongside escalating resource depletion and ecological degradation, underscores the urgent need for innovative paradigms in urban development. In response, sustainable smart cities are increasingly leveraging advanced technological frameworks—most notably the convergence of Artificial Intelligence of Things (AIoT) and Cyber-Physical Systems (CPS)—as critical enablers for transforming their management and planning processes. Within this dynamic landscape, *Urban Brain* (UB) and *Urban Digital Twin* (UDT) have emerged as prominent AIoT-powered city platforms. Defined by their complex functionalities and multi-layered architectures, these systems exemplify *Cyber-Physical Systems of Systems* (CPSoS), offering a cohesive foundation for integrating real-time operational responsiveness with strategic predictive foresight. Despite notable technological progress, a critical gap persists in effectively integrating the distinct yet complementary capabilities of UB and UDT within a structured and scalable framework. To the best of our knowledge, research on the explicit fusion of UB's real-time analytics—enabled through stream processing—with UDT's predictive analytics—driven by simulation modeling—is scant, if not absent. Most existing studies continue to treat UB and UDT as siloed systems, failing to recognize the critical need to synchronize their respective operational and strategic functions. This fragmentation limits the ability of urban systems to respond both adaptively and proactively to the complex, interrelated challenges of environmental sustainability. To address this critical gap, this study introduces a novel foundational framework—Artificial Intelligence of Things for Sustainable Smart City Brain and Digital Twin Systems—designed to synergistically integrate UB and UDT as AIoT-enabled platforms within a unified CPSoS architecture. This framework addresses the critical disconnect between real-time operational management and strategic predictive planning, delivering an integrated pathway for advancing environmentally sustainable smart city development goals. Harnessing the complementary strengths of UB and UDT, it empowers cities to respond dynamically to immediate urban demands while ensuring consistent alignment with long-term sustainability goals. UB's real-time analytics enhance the efficiency of daily urban operations, whereas UDT's predictive modeling anticipates and simulates future scenarios. Together, they establish a synergistic feedback loop: UB's real-time insights continuously inform UDT's strategic simulations, while UDT's long-range forecasts iteratively refine UB's operational decision-making. The framework thus equips researchers, practitioners, and policymakers with a robust methodology for designing and implementing adaptive, efficient, and resilient urban ecosystems. It facilitates the development of intelligent urban environments that can advance environmental sustainability by integrating solid theoretical foundations with actionable strategies.

## Highlights

• The AIoT and CPSoS fusion is a transformative force driving innovation in sustainable smart city management and planning.
•UB and UDT exemplify AIoT-enabled CPSoS for sustainable smart city operational functioning and strategic planning.
•The proposed framework integrates UB and UDT to advance environmentally sustainable smart city development goals.
•UB's real-time insights guide UDT's simulations, while UDT's predictive forecasts enhance UB's operational decisions.
•The framework empowers stakeholders to design and implement adaptive, efficient, and resilient urban ecosystems.

## Graphical abstract

**Figure:**
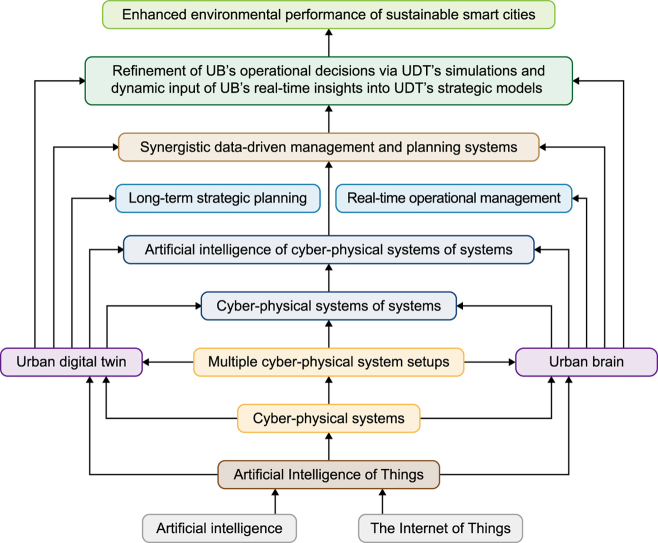

## 1 Introduction

The rapid pace of urbanization has intensified the challenges of environmental sustainability, with resource depletion, ecological degradation, and excessive carbon emissions becoming critical global concerns. As urban areas continue to expand and demand more resources, the importance of adopting cutting-edge technologies and innovative strategies to tackle these multifaceted challenges has grown significantly. Sustainable smart cities embody a transformative vision for urban development, harnessing advanced solutions to address the intricate challenges of modern urbanization and its far-reaching impacts. While this emerging urbanism paradigm emphasizes the environmental, economic, and social dimensions of sustainability (e.g., Ref. [[1], [2], [3], [4], [5]]), in recent years, its focus has increasingly shifted towards addressing the pressing challenges of environmental sustainability and mitigating the impacts of climate change [6,7]. This shift, initially driven by the adoption of IoT and Big Data technologies, has evolved to integrate green technologies and data-driven innovations, fostering more adaptive, efficient, resilient, and sustainable urban solutions (e.g., Ref. [[8], [9], [10], [11], [12]]).

In more recent years, both smart cities and sustainable smart cities have notably embraced and harnessed more advanced technologies, particularly Artificial Intelligence (AI) [[13], [14], [15], [16], [17], [18], [19]], Artificial Intelligence of Things (AIoT) (e.g., Ref. [[20], [21], [22], [23]]), and Cyber-Physical Systems (CPS) (e.g., Ref. [[24], [25], [26], [27], [28]]) to enhance energy efficiency, reduce resource consumption, mitigate carbon emissions, and build climate resilience [6,25,29]. These technologies are reshaping urban systems and driving the future of sustainable urban development by enabling innovative solutions across environmental and climate domains (e.g., Ref. [25,[30], [31], [32], [33], [34], [35], [36], [37], [38], [39], [40], [41], [42], [43]]). Through their integration, AIoT and CPS provide powerful capabilities for data-driven decision-making, real-time optimization, predictive modeling, intelligent automation, adaptive system control, and dynamic resource allocation, which play a critical role in advancing the environmental goals of sustainable smart cities. Through these integrated innovations, sustainable smart cities are equipped to tackle immediate operational management challenges effectively while laying a foundation for strategic planning and sustainability goals.

AIoT, which integrates IoT infrastructure and connectivity with AI's computational and analytical capabilities, plays a key role in addressing complex energy and environmental issues across urban and industrial systems in smart cities and beyond (e.g., Ref. [6,35,[44], [45], [46], [47], [48], [49], [50]]). AIoT integrates the data generation capabilities of IoT with the advanced algorithmic power of AI, enabling real-time data processing, advanced analytics, adaptability to dynamic conditions, and intelligent decision-making. CPS complements these capabilities by operationalizing insights to dynamically monitor, control, and optimize urban systems in real-time. This synergistic relationship is foundational. AIoT delivers the deep insights required for sophisticated urban and industrial solutions, while CPS enhances AIoT's effectiveness by offering real-time feedback loops and adaptive mechanisms (e.g., Ref. [[51], [52], [53], [54]]). These technologies significantly improve the efficiency and functionality of urban systems by establishing a cohesive ecosystem for urban management and planning, ensuring their alignment with environmental sustainability objectives [25,55]. Their synergy is particularly pronounced in the context of smart cities, where integrated frameworks enable real-time monitoring, predictive planning, and adaptive responses across various domains. Within this integrated ecosystem, CPS plays an important role by enhancing the efficiency and management of key urban domains, including transportation, energy, air pollution, waste, and water systems, while also addressing critical intersections such as the water-energy-food-climate nexus in smart city systems [41,[56], [57], [58]].

In light of the above, the integration of AIoT and CPS amplifies the transformative capabilities of emerging city platforms for data-driven management and planning. Among the most prominent are Urban Brain (UB) (e.g., Ref. [[59], [60], [61], [62]]) and Urban Digital Twin (UDT) [[63], [64], [65], [66], [67], [68]], which exemplify and demonstrate the seamless integration of AIoT and CPS technologies (e.g., Ref. [32,[69], [70], [71], [72], [73], [74]]) to propel the development of sustainable smart cities, particularly in addressing critical environmental challenges through advanced data-driven management and planning ([25,55], e). UB and UDT provide advanced frameworks for achieving urban efficiency and ecological resilience by combining the real-time analytical power of AIoT with the integrated operational capabilities of CPS.

UB leverages AI-driven real-time analytics to optimize urban management operations, focusing on critical areas such as traffic, transportation, energy, pollution, and public safety [61,62,[74], [75], [76], [77]]. Meanwhile, UDT harnesses AI or AIoT capabilities to create dynamic, high-fidelity digital replicas of urban environments, facilitating scenario testing, predictive modeling, and strategic planning [37,[63], [64], [65], [66], [67],^73^]. These capabilities enable cities to anticipate future challenges and design effective, sustainable interventions. AIoT-driven UDT provides a comprehensive framework for advancing sustainable smart city planning by addressing key aspects of environmental sustainability, such as resource allocation, traffic prediction, infrastructure development, energy forecasting, renewable energy deployment, climate scenario analysis, biodiversity preservation, pollution monitoring and mitigation, and waste and water management strategies (see Bibri et al. [64] for an in-depth review). Together, UB and UDT are increasingly recognized as indispensable components of large-scale AIoT-CPS systems for sustainable smart cities, addressing the complexities of environmental sustainability and providing advanced capabilities for integrated, dynamic management and adaptive planning processes. AIoT powers the intelligence layer of CPS by securely analyzing vast datasets using Machine Learning (ML) and Deep Learning (DL) techniques [[78], [79], [80], [81]] to deliver actionable insights for resource optimization, energy efficiency, environmental monitoring, climate modeling, and resilience building [6,45,82,83].

Furthermore, CPS focuses on single, self-contained systems designed to optimize specific tasks within urban domains, such as localized traffic flow, smart grids, autonomous vehicles, and water distribution networks. In contrast, Cyber-Physical Systems of Systems (CPSoS) [84,85], such as UB and UDT encompass multiple CPS setups working collaboratively to achieve system-level objectives in real-time (e.g., Refs. [25,25,[59], [60], [61],^64^,^74^,^86^,^87^,^88^]). UB and UDT should be treated as CPSoS due to their larger scale, heightened complexity, and more demanding requirements compared to the recent applications of other types of DTs (e.g., Ref. [25,66,67,71,89,90]), which facilitate the integration of physical and digital components across a wide array of domains [91]. The integration of UB and UDT as a unified CPSoS is particularly impactful, as it enables sustainable smart cities to operate dynamically and address complex environmental challenges.

This study focuses on the integration of UB and UDT systems in the context of AIoT-enabled CPSoS to support environmentally sustainable urban development. The scope encompasses both real-time operational management (via UB) and strategic predictive planning (via UDT), emphasizing their roles in achieving cross-domain coordination, dynamic responsiveness, and predictive foresight. Despite their collaborative and synergistic potential, UB and UDT are often developed and deployed in isolation, leading to fragmented data flows, suboptimal decision-making, and misaligned urban strategies.

While significant advances in UB and UDT, driven by the convergence of AIoT and CPS, have been achieved, a critical gap remains in integrating their distinct yet complementary capabilities within a unified framework. To the best of our knowledge, research that explicitly explores the fusion of UB’s real-time analytics, enabled through stream processing, with UDT’s predictive analytics, driven by simulation modeling, is scant, if not absent, in the existing literature. Existing research largely treats UB and UDT as siloed systems, overlooking the critical need to synchronize their management and planning functions. The central problem this study addresses is the absence of a cohesive and scalable framework capable of synchronizing UB’s real-time insights with UDT’s simulation-driven foresight. This fragmentation hampers the ability of sustainable smart cities to holistically manage complex systems and respond effectively to interconnected environmental challenges. It highlights the strategic importance of integrated models that enable cities to balance operational demands with strategic objectives. As corroborated by Webb et al. [92], “urban policy and decision makers are challenged by the complexity of cities as social-ecological–technical systems. Consequently, there is an increasing need for collaborative knowledge development that supports a whole-of-system view and transformational change at multiple scales. Such holistic urban approaches are rare in practice … Developing integrated strategies at broader urban scales is seen as the most pressing need.”

To address these critical gaps, this study introduces a pioneering foundational framework—Artificial Intelligence of Things for Sustainable Smart City Brain and Digital Twin Systems—which synergistically integrates UB and UDT as AIoT-driven CPSoS. This unified framework fosters a cohesive, intelligent urban ecosystem by breaking down the silos between prominent city platforms and enabling a holistic approach to sustainable urban development. It supports both real-time operational responsiveness and strategic predictive foresight, dynamically aligning immediate actions with broader goals in the context of environmental sustainability. Designed as a practical and scalable structure, the framework enables seamless integration of real-time monitoring, adaptive control, and predictive analytics to support more responsive and foresight-driven urban management. In contrast to conceptual models that focus largely on theoretical constructs, this study offers a functionally integrated, operational model with direct relevance for urban practitioners and policymakers working to advance environmentally sustainable smart cities. The central research question guiding this inquiry is:

How can UB's real-time operational management and UDT's strategic predictive planning functions be integrated into a unified AIoT-driven CPSoS framework to enhance the environmental performance of sustainable smart cities?

To address this research question, the study pursues the following four objectives.

(1). To establish the theoretical and practical foundations of the proposed framework by exploring the recent environmental shift towards sustainable smart cities, the role of AIoT in urban management and planning, and the advancements in CPS, CPSoS, and Artificial Intelligence for the Internet of Everything (AIoE), as well as their convergences with UB and UDT.
(2). To examine the architectural and functional dimensions of UB and UDT, emphasizing their synergistic operation within AIoT-driven ecosystems.
(3). To evaluate the contributions of UB and UDT to data-driven urban management and planning, with a particular focus on their impact on the critical domains of environmental sustainability.
(4). To develop a unified AIoT-driven CPSoS framework that synergistically integrates UB's real-time operational management processes with UDT's strategic predictive planning functions*.*

This study makes several significant contributions to the growing discourse on sustainable smart cities. First, it introduces a unified, scalable framework that bridges the gap between real-time operational management and strategic predictive planning, offering actionable solutions to optimize urban resource allocation, reduce emissions, and enhance resilience by focusing on interoperability, scalability, and coordination. Accordingly, it contributes both theoretically and practically to advancing intelligent, adaptive, and data-driven environmental management and planning in sustainable smart cities. Second, the foundational framework leverages the complementary strengths and synergistic capabilities of UB and UDT, demonstrating how AIoT-powered CPSoS can dynamically respond to immediate urban challenges while simultaneously anticipating and mitigating long-term impacts. UB's real-time analytics enhance the operational efficiency of urban systems, while UDT's predictive capabilities provide strategic optimization for long-term sustainability. For example, within the CPSoS framework, energy management demonstrates the seamless integration of operational efficiency and strategic foresight. UB dynamically optimizes energy distribution in real-time in smart grids, preventing overloads and ensuring efficient usage, while UDT forecasts energy demand patterns and develops strategies for integrating renewable energy sources. This integration aligns real-time energy management with long-term sustainable energy goals, enhancing energy efficiency, maximizing the utilization of renewable resources, minimizing energy losses, strengthening system resilience, and achieving significant cost savings.

Third, the study underscores the transformative role of AIoT as a versatile enabler and unifying technological paradigm for integrating and coordinating multiple CPS setups within UB and UDT platforms. The framework establishes a blueprint for integrating other city platforms and urban domains by facilitating cross-system communication, ensuring seamless interoperability, and fostering cohesive urban strategies across multiple domains. This integration enhances the overall functionality and scalability of urban systems, ensuring they remain dynamic and responsive to evolving environmental challenges while aligning short-term actions with broader sustainability objectives. Finally, the study equips stakeholders with both theoretical and practical approaches to designing and implementing adaptive, efficient, and resilient urban ecosystems. It offers a holistic approach with actionable solutions to advance environmental sustainability goals by integrating dynamic management with adaptive planning in smart cities.

The remainder of this study is structured as follows: Section 2 provides a survey of related work, situating the study within the broader research landscape. Section 3 outlines the methodology applied in analyzing and developing the framework. Section 4 presents a comprehensive thematic literature review that forms the foundation for developing the foundational framework. Section 5 details the framework by describing its components, processes, interconnections, and derivation process. Section 6 presents a comprehensive discussion, encompassing a summary of the findings and their interpretation, implications, challenges, limitations, and suggestions for future research.Section 7 concludes the study by summarizing key points, outlining the primary contributions, and highlighting the main takeaways. This provides a clear recap of the research's significance and implications for the field.

## 2 Related work

Recent advancements in AIoT, CPS, UB, and UDT technologies have significantly reshaped data-driven environmental urban management and planning, particularly in the context of sustainable smart cities. These technologies enhance operational efficiency, facilitate predictive planning, and foster more resilient urban environments. Despite extensive research on these technologies, a gap remains in integrating UB and UDT within a unified AIoT-driven CPSoS framework. This section addresses this gap by reviewing existing conceptual frameworks and models for Next-Generation CPS, UDT, and UB, with a focus on large-scale AIoT-driven urban systems. While many frameworks have been proposed for these technologies, the integration of UB and UDT as CPSoS—especially as interconnected city platforms within AIoT ecosystems—has largely been unexplored. This review critically examines several relevant frameworks, highlighting their design principles, contributions, and practical applications while also identifying areas that require further research. Ultimately, this analysis underscores the need for the innovative framework introduced in this study.

Mishra et al. [38] proposed a Next-Generation CPS framework that integrates IoT, AI, and big data analytics to address smart city challenges. While this framework provides a comprehensive view of smart city technologies, it does not focus on the integration of specific CPS-enabled urban platforms, such as UB and UDT. It does not explore the potential for cohesive urban systems that can optimize planning and governance. Although the framework offers a holistic design, the lack of attention to real-time and predictive integration is a significant gap in the context of AIoT and sustainable urban management and planning.

Bibri et al. [93] reviewed the intersection of AIoT, AI, and UDT, emphasizing the integration of these technologies for advancing sustainable smart cities. Their framework focuses on environmental planning by combining AIoT's data analytics with UDT's simulations. However, this study primarily targets UDT's application in isolation and overlooks opportunities for broader integration with other large-scale AIoT-driven systems. This focus limits the framework's capacity to foster a more interconnected approach to urban management and planning. Bibri et al. [64] expanded on their previous work by introducing a framework that integrates UB, Smart Urban Metabolism (SUM), and platform urbanism for sustainable city development. The framework highlights the value of real-time data analytics and predictive modeling but fails to address the integration of advanced city modeling and simulation, which is crucial for enabling broader synergies across urban management systems. The absence of an integrated approach to UB, UDT, and other platforms limits the practical applicability of the proposed framework.

Zarrabi and Doost Mohammadian [67] investigate the integration of DT, IoT, and AI in urban intelligence, outlining their roles in enhancing sustainability, resilience, and governance. Their study synthesizes insights from various theoretical frameworks, enhancing the understanding of how the fusion of these technologies can optimize urban systems and contribute to the development of more sustainable and resilient smart cities. However, while the study involves different CPS setups concerning both data-driven urban management and planning, it does not address their integration within the broader context of AIoT-driven CPSoS, particularly in the context of dynamic, interconnected urban environments.

Wang et al. [87] proposed an the Internet of DT (IoDT) architecture for real-time synchronization and dynamic mission coordination across urban systems. While the study provides valuable insights into security and privacy challenges, it does not address the potential of AI to enhance the computational and analytical capabilities of IoDT systems or explore its role in urban planning. The narrow focus on technical architecture neglects the broader implications of AIoT-driven frameworks for integrated urban management or planning. Exploring the UB system as a conceptual framework for smart cities, Liu et al. [94,95] highlight the need for a unified technology framework to improve urban management. Although their work is foundational in understanding the theoretical principles behind City Brain, it does not address its integration with other city platforms. This lack of operational integration limits the ability of UB systems to enhance urban management and decision-making.

While these studies contribute significantly to the development of AIoT, CPS, UB, and UDT technologies, they remain fragmented in their approach to integrating these systems into cohesive urban management and planning frameworks. The absence of a unified, scalable framework that synergizes UB's real-time monitoring and responsiveness with UDT's predictive modeling and strategic foresight underscores a critical gap in current research. To address this gap, future research must explore how to integrate UB and UDT as interconnected components within a larger AIoT-driven CPSoS framework, which can more effectively address the dynamic and interconnected challenges of environmental sustainability in smart cities. This knowledge gap underscores the need for a comprehensive framework that leverages the complementary strengths of UB, UDT, and AIoT to develop an integrated solution for environmentally sustainable urban management and planning.

## 3 Research methodology

A thematic literature review is a structured and systematic methodology for examining existing research, focusing on identifying, analyzing, and synthesizing recurring themes, patterns, and relationships within the literature. This approach is particularly well-suited for interdisciplinary topics, including the integration of AIoT, CPSoS, UB, and UDT for sustainable smart city development, where diverse perspectives must be unified into a coherent narrative. It highlights key trends, synthesizes critical insights, and identifies gaps in the current knowledge base by organizing the body of work into central themes and categories.

In the context of this study, the thematic literature review builds on and further substantiates the gaps identified in the previous section, particularly the absence of comprehensive frameworks that integrate UB and UDT as AIoT-driven CPSoS for sustainable smart city management and planning. This review provides a robust foundation for developing a comprehensive framework that aligns operational and strategic capabilities, addressing the complexities of environmental sustainability and urban resilience. Categorizing prior studies under thematic headings enables the identification of synergies, disconnects, and priority areas for improvement, ensuring that both the strengths and limitations of existing research inform the foundational framework.

This structured methodology integrates and fuses findings from different disciplines, creating a unified narrative that addresses the identified gaps and advances the understanding of how emerging technological paradigms can support sustainable smart cities in improving their contribution to environmental goals. The thematic literature review substantiates the relevance and originality of the proposed framework by synthesizing insights from the latest research and development works. Furthermore, it provides a systematic lens for exploring the interplay of advanced technologies in achieving integrated dynamic management, adaptive planning, and long-term sustainability objectives.

As part of the thematic literature review, the research design incorporated a curated selection of case studies to provide empirical evidence. Case studies on UB and UDT were synthesized to gain practical insights into their technological complexities, functional dynamics, and contributions to environmental sustainability. These case studies were chosen based on their relevance to the interconnected fields of data-driven urban management and planning, offering a grounded understanding to inform and refine the proposed framework.

**Fig. 1:**
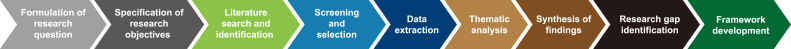
A flow diagram outlining the step-by-step process of developing the foundational framework based on the insights derived from the thematic literature review.

The thematic literature review followed a structured and systematic process consisting of nine stages (Fig. 1). This multi-phase approach ensured a comprehensive examination of the existing literature, providing a robust foundation for identifying synergies and opportunities in synergizing UB and UDT as AIoT-driven CPSoS in the context of sustainable smart cities.

**Fig. 2:**
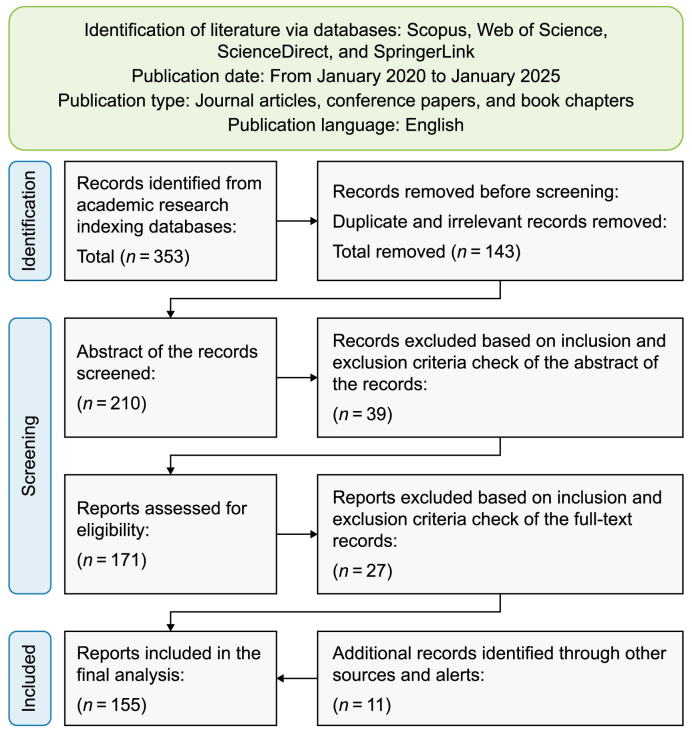
The PRISMA flowchart for literature search and selection. Adapted from Page et al. [96].

Fig. 2 illustrates the Preferred Reporting Items for Systematic Reviews and Meta-Analyses (PRISMA) approach adopted for the literature search and selection process. The approach involved utilizing leading academic research indexing databases, including Scopus, Web of Science (WoS), ScienceDirect, and SpringerLink, known for their extensive coverage of high-quality peer-reviewed studies. These databases were selected to ensure comprehensive retrieval of scholarly works pertinent to the multifaceted topic addressed in this study. To enhance specificity and relevance, the literature search was guided by carefully curated keywords and their combinations, which were systematically organized into thematic clusters or categories. These clusters were designed to capture the breadth and depth of existing knowledge while aligning with the study's interdisciplinary focus.

The selection of keywords reflected the multidimensional nature of this research and ensured a thorough exploration of related literature across various fields. These keywords included: “Smart Cities,” “Sustainable Smart Cities,” "Cyber-Physical Systems," "Cyber-Physical Systems of Systems," "Urban Digital Twin," "City Brain," "Artificial Intelligence of Things," "Internet of Everything," “Artificial Intelligence of the Internet of Everything,” “Environmental Management,” and “Environmental Planning.” Boolean operators were employed to refine search results and ensure the inclusion of studies addressing multiple intersecting themes. These combinations allowed for a precise yet comprehensive review of the literature. Examples of keyword combinations included: ("Cyber-Physical Systems" OR "Cyber-Physical Systems of Systems") AND ("Smart Cities" OR "Sustainable Smart Cities"); (Artificial Intelligence" OR "Artificial Intelligence of Things") AND ("Smart Cities" OR "Sustainable Smart Cities"); ("Digital Twin" OR "Urban Digital Twin") AND ("Artificial Intelligence” OR “Artificial Intelligence of Things"); ("Digital Twin" OR "Urban Digital Twin") AND ("Smart Cities" OR "Sustainable Smart Cities”); (“Urban Brain” OR “City Brain”) AND (“Smart Cities” OR "Urban Management"); and ("Artificial Intelligence of Things" AND "Environmental Sustainability") OR (“Artificial Intelligence of Things” AND “Sustainable Development”). These keywords and their combinations were applied to search the title, abstract, and keyword fields of articles, generating preliminary insights into the relevant body of literature.

The thematic and systematic approach ensured that the literature search captured the diverse and interconnected aspects of the study, providing a robust foundation for analyzing the state-of-the-art sustainable smart city technologies for data-driven management and planning. The combination of thematic keyword clusters and Boolean logic facilitated the identification of both broad trends and nuanced insights in the existing body of literature.

The inclusion and exclusion criteria outlined in Table 1 were carefully designed to ensure a focused and targeted selection of studies. This process emphasizes high-quality, impactful, peer-reviewed research that aligns with the study's objectives, while filtering out irrelevant or insufficiently detailed works.

**Table 1:** Inclusion and exclusion criteria.

Inclusion Criteria	Exclusion Criteria
Articles focusing on two or more of these themes: CPS, CPSoS, AIoT, AIoE, UB, and UDT.	Studies focusing exclusively on unrelated fields, such as theoretical AI models or non-urban AIoT applications.
Studies explicitly discussing the integration of these technologies or frameworks in smart city management and planning.	Duplicate studies identified across databases were excluded during the screening process.
Research highlighting technological synergies, computational processes, or integration frameworks in AIoT systems within smart cities or sustainable smart cities.	Articles that do not address the interplay or synergy between the core technologies and concepts.
Articles exploring AI- or AIoT-driven approaches to sustainable development or environmental sustainability in smart cities or sustainable smart cities.	Research lacking empirical evidence or comprehensive analysis relevant to the study objectives.
Peer-reviewed journal articles, conference papers, or book chapters published in reputable sources.	Articles published before 2020 unless deemed foundational or heavily cited in the domain.
Studies published between 2020 and 2025 were included to ensure the inclusion of recent advancements and emerging trends.	Grey literature, opinion pieces, editorials, or non-peer-reviewed conference abstracts.
Articles published in English to ensure consistency in analysis and interpretation.	Articles not published in English could pose challenges for consistent analysis and interpretation.

The timeframe of 2020–2024 was selected to ensure that the review captures the most recent advancements and emerging trends in the rapidly evolving domains of AIoT, CPS, CPSoS, UB, and UDT, as well as their integration in the context of smart cities. Given the exponential growth of research in sustainable smart city technologies and the accelerated convergence of these technological paradigms or city platforms in recent years, this span prioritizes cutting-edge studies while maintaining relevance to current technological and theoretical developments. This focus on contemporary literature aligns with the study's objective to develop a foundational framework informed by the latest innovations and applications.

The data extraction process involved gathering relevant information from the selected studies to ensure consistency and accuracy. Key details, including study objectives, findings, and thematic insights, were recorded. A structured approach was employed to systematically capture essential attributes, including core concepts, technologies, paradigms, application domains, integration frameworks, and identified challenges. This organized approach facilitated the collation of comprehensive and comparable data, enabling a thorough evaluation of each study's relevance to and alignment with the research objectives.

The analysis phase involved a critical review of the extracted data to identify recurring themes, patterns, and relationships across the studies. Thematic analysis was employed to group findings into pre-defined and emergent categories, focusing on technological synergies, integration challenges and opportunities, and contributions to sustainable smart city management and planning. Special attention was given to examining the interplay between AIoT, CPSoS, UB, and UDT, as well as their role in addressing urban complexities such as environmental sustainability and urban resilience. The analysis also involved cross-comparing studies to assess their alignment with the research objectives and identify areas of convergence and divergence.

The synthesis process integrated the analyzed findings into a coherent narrative that informs the development of the foundational framework. Insights from thematic categories were combined to highlight synergies, address gaps, and identify opportunities for future advancements. The synthesis emphasized the role of AIoT-driven CPSoS in aligning operational management efficiencies with strategic urban planning and environmental goals. It created a unified understanding of how emerging technologies can be effectively leveraged to foster sustainable smart city development by drawing connections between diverse studies. The resulting narrative reflects the state-of-the-art in the field and substantiates the originality and relevance of the proposed framework.

## 4 A comprehensive state-of-the-art thematic literature review

This section reviews the foundational concepts and practical advancements underpinning the study, focusing on the recent shift of sustainable smart cities towards addressing environmental sustainability, the transformative role of AI and AIoT in advancing environmental goals, and the nuanced distinction between CPS and CPSoS. This distinction highlights their technical components and capabilities in operational management and strategic planning of urban systems, illustrating the synergistic interplay between UB and UDT as either multifaceted CPS setups or forms of CPSoS. The interconnected functions of UB and UDT, previously introduced and linked to these concepts in the introduction, are further contextualized within the theoretical and practical foundations to support the development of the proposed framework. Furthermore, this section highlights the transformative interplay of AIoE, CPSoS, and DTs as critical paradigms shaping data-driven urban management and planning. It provides a comprehensive understanding of how these technologies converge to advance environmental goals in sustainable smart cities by examining the dynamic interconnections between AIoE and CPSoS, as well as the integration of DTs into CPS and CPSoS. This synthesis culminates in an integrative analysis, offering insights into the operational synergies and transformative capabilities of these paradigms in fostering resilient, efficient, and adaptive urban ecosystems.

### 4.1 Theoretical and practical foundations

#### 4.1.1 Sustainable smart cities: environmental sustainability and climate Resilience

Sustainable smart cities have emerged as an integrated model for urban development, emphasizing the convergence of advanced technologies and innovative practices to address complex urban challenges. These cities are designed to optimize resource utilization, enhance energy efficiency and conservation, reduce carbon emissions, and build resilience against climate change. Fundamentally, these urban environments are designed to advance the environmental, economic, and social pillars of sustainability, aligning with overarching goals such as the Sustainable Development Goals (SDGs) (e.g., Ref. [1,2,4,97,98]). However, due to the pressing need to combat ecological degradation, resource depletion, and climate instability, there has been a shift toward prioritizing the environmental dimension of sustainability (see Bibri et al. [7] for a bibliometric analysis). This evolving focus has positioned emerging technologies such as AI [13,15,17,[99], [100], [101]] and AIoT [6,20,22,30,35,37,42,102] at the forefront of sustainable urban and environmental innovation. Especially in urban areas, which are significant contributors to greenhouse gas emissions and resource consumption, have become focal points for advancing environmental sustainability goals through advanced technological solutions.

The evolution of sustainable smart cities relies heavily on advanced technologies to address complex environmental challenges and bolster resilience. Several recent studies have highlighted the innovative role of AI and AIoT in addressing climate change and promoting environmental sustainability, positioning them as cornerstones of sustainable urban development. These works underscore the multifaceted applications of these emerging technologies in advancing integrative and adaptive strategies essential for sustainable smart cities. Bibri [6] offers a comprehensive review of the evolving landscape of sustainable smart cities and smarter eco-cities, emphasizing the transformative impact of applied AI and AIoT solutions. The study highlights their critical role in advancing environmental sustainability and driving climate action. The findings reveal the significant potential of these technologies to optimize resource utilization, improve infrastructure development, facilitate renewable energy deployment, monitor and analyze environmental conditions, reduce carbon emissions, enhance air and water quality, promote sustainable transportation, support biodiversity conservation, advance waste, and water management, and strengthen climate resilience. Despite their promising opportunities, the study emphasizes that AI and AIoT face technical, ethical, social, regulatory, and environmental challenges—including data security and privacy, bias and fairness, transparency and accountability, energy and water consumption, and data governance—that must be effectively addressed to ensure their successful implementation.

In the realm of AI, Sahil et al. [103] highlight the critical role of AI in mitigating climate change through advanced predictive tools and data-driven interventions. Their study highlights how AI can contribute to achieving the United Nations' SDG 13, “Climate Action”, by reducing greenhouse gas emissions, predicting extreme weather events, and facilitating climate-related time series analysis. Shaamala et al. [104] focus on optimizing Green Infrastructure (GI) using AI to address climate change. Their systematic exploration identifies key areas of GI optimization, such as air quality, biodiversity, energy efficiency, and urban heat island mitigation. The study also introduces a framework to guide AI-driven GI optimization processes within the context of climate adaptation and mitigation. Nti et al. [100] explore AI applications in environmental sustainability, particularly in biodiversity, energy, transportation, and water management. They discuss how AI technologies, such as neural networks, Expert Systems (ES), and Computer Vision (CV), are employed to predict and optimize ecosystem services, conserve water resources, enhance energy efficiency, and improve transportation systems. These insights collectively illustrate AI's innovative role in shaping sustainable smart cities capable of addressing pressing environmental challenges.

#### 4.1.2 Artificial intelligence of things for environmental management and planning in sustainable smart cities

With the recent groundbreaking convergence of AI and IoT, sustainable smart cities are particularly leveraging AIoT as a transformative technological paradigm. This enables the development of intelligent systems capable of processing vast amounts of real-time data to make informed decisions, implement adaptive measures, and execute strategic interventions (e.g., Ref. [20]; Alwar et al., 2025; [23,35,39,102,105]). AI and AIoT are being utilized not only to address environmental management challenges (e.g., Ref. [6,22,25,25,[44], [45], [46], [47], [48], [49]]) but also to enhance environmental planning strategies (e.g., Ref. [33,55,64,106]; Kamrowska-Załuska et al., 2021; [[107], [108], [109]]). In urban management, AIoT enables real-time monitoring and control systems, ensuring that operations are dynamically optimized to maximize efficient resource utilization. In urban planning, AIoT facilitates strategic decision-making through simulations, scenario modeling, and predictive analytics, which help restructure and redesign sustainable and resilient urban environments, as well as develop newly planned urban districts or areas.

AIoT provides the foundation for creating cities that are both operationally efficient and environmentally sustainable by enabling intelligent and adaptive systems. This dual capability bridges operational functioning and strategic planning, creating an integrated framework that enhances the adaptive and dynamic capacities of urban systems. Kataria et al. [35] examine the innovative potential of AIoT in reshaping urban planning and management, emphasizing its critical role in advancing sustainable smart cities. AIoT enables intelligent systems to perform real-time data collection, analysis, and decision-making, driving innovative applications. While still an emerging technology, AIoT presents significant opportunities to enhance dynamic management and improve resilience planning, thereby contributing to the development of next-generation smart cities. Likewise, Alwar et al. [30] examine how the integration of IoT and techn AI ologies can support the development of sustainable smart cities. Using case studies from cities such as Singapore, Barcelona, and Copenhagen, the study showcases the application of IoT-AI solutions—including smart grids, adaptive traffic control, predictive waste management, and AI-based surveillance—to improve urban efficiency and sustainability. The research reports notable gains, including a 25 % increase in energy efficiency, a 30 % reduction in traffic congestion, and a 40 % improvement in waste management. These findings highlight the transformative potential of IoT and AI in creating more resilient, efficient, and livable urban environments.

AIoT is transforming urban sustainability by facilitating seamless cross-domain integration and fostering holistic strategies. Bibri [29] explores the catalytic role of the convergence of AI and IoT in advancing the development of smarter eco-cities. The study focuses on leveraging the synergies of circular economy, metabolic circularity, and tripartite sustainability to align smarter eco-cities with SDGs. The findings highlight that AIoT capabilities support circular economy principles, such as resource regeneration and waste minimization, and optimize urban metabolic circularity by improving the flow, reuse, and closed-loop systems of materials and energy. Moreover, AIoT facilitates tripartite sustainability by balancing environmental, economic, and social dimensions, fostering a holistic approach to urban development. In a nutshell, AIoT enables smart city systems to adapt dynamically to evolving challenges, fostering operational optimization and long-term sustainability by integrating real-time data analytics with advanced decision-making capabilities.

Despite the future prospects of AIoT, Alwar et al. [30] identify major adoption barriers—such as data privacy, interoperability issues, and high costs—and propose a governance and infrastructure framework to support scalable and secure implementation. Similarly, as Kataria et al. [35] point out, several challenges must be addressed for the effective implementation of sustainable smart city management and planning. These include the need for secure and comprehensive data management systems to safeguard vast amounts of collected data against unauthorized access and misuse, as well as developing unbiased AI algorithms, which is critical to ensure equitable outcomes. The training data must accurately represent the demographics of the populations the AI systems aim to serve. The authors underscore that, although these challenges persist, the future of AIoT in sustainable urban development remains promising. Advancements in the field are expected to yield more innovative and effective applications, further enhancing the sustainability and functionality of urban infrastructures. The study calls for continued research and development to overcome these barriers and unlock the full potential of AIoT in creating smarter, more sustainable cities.

#### 4.1.3 Models and techniques in artificial intelligence of things

AIoT utilizes various models and techniques from AI subdomains, especially ML, DL, CV, and Natural Language Processing (NLP), particularly in the context of smart cities (e.g., Ref. [31,39,42,50,53,110,111]). ML models, such as Random Forests, Support Vector Machines (SVM), Gradient Boosting, and *k*-nearest Neighbors (k-NN), are used for predictive analytics, anomaly detection, and optimization of processes such as energy consumption, air quality monitoring, and waste management. Similarly, DL models, such as Convolutional Neural Networks (CNNs), Recurrent Neural Networks (RNNs), and Long Short-Term Memory Networks (LSTMs), excel in pattern recognition, climate modeling, and resource allocation tasks. CV models enable systems to monitor and analyze visual data, contributing to applications such as waste monitoring, traffic analysis, and biodiversity assessment, thereby improving operational efficiency and reducing environmental impacts. NLP contributes to AIoT by facilitating human-machine interactions, analyzing sentiment for citizen engagement, and processing textual data from urban reports, policy documents, and sensor-generated alerts.

**Fig. 3:**
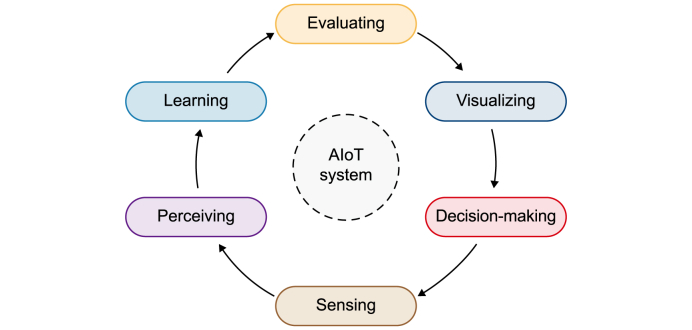
Six pillars of Artificial Intelligence of Things systems [112].

Furthermore, AIoT systems operate within a structured framework (Fig. 3) composed of a set of interdependent components, typically ranging from four to six, depending on the specific application context. These include processes such as perceiving, learning, reasoning, and behaving [111]; sensing, perceiving, learning, visualizing, and decision-making [22]; and sensing, perceiving, learning, evaluating, visualizing, and acting [112]. These layered functions are foundational to advancing urban environmental management and planning, as they enable more adaptive, data-driven, and responsive decision-making in complex urban systems.

AIoT is reshaping critical domains by enabling intelligent, autonomous, and sustainable solutions. Recent research highlights the diverse AI technologies, approaches, and application areas that collectively drive innovation within AIoT ecosystems (Table 2).

**Table 2:** Key relevant studies on models, techniques, approaches, and applications in Artificial Intelligence of Things ecosystems.

Application Area	AI Models and Techniques	Specific Approaches	Relevant Applications in AIoT	Citation
Environmental sustainability and climate resilience	ML, DL, NLP, CV, IoT platforms	Predictive analytics, autonomous decision-making	Renewable energy optimization, water resource management, smart agriculture, environmental monitoring, sustainable urban development, climate resilience	Thamik et al. [42]
Smart cities and urban management	ANN, CNN, LSTM, SVM	AI algorithms for automation and efficiency	Waste management, mobility solutions, risk management, urban security, smart city management	Anjum et al. [31]
Sustainable development and infrastructure innovation	ML, DL, CV, NLP	Real-time analytics, predictive modeling, federated learning, and blockchain-enabled security	Environmental monitoring, smart infrastructure, smart grids, precision agriculture, energy management, urban planning, climate resilience	Mishra et al. [39]

These studies collectively demonstrate the strategic role of AI models, advanced AIoT frameworks, and emerging technologies in delivering intelligent and sustainable solutions. AIoT facilitates achieving sustainability goals, enhances urban resilience, and transforms infrastructure management in sustainable smart environments.

Models and techniques from ML and DL are most extensively applied in smart cities and environmental sustainability, as well as within AIoT frameworks, for their capability to process vast amounts of environmental and climate data in real-time (e.g., Ref. [6,22,30,35,42,99,101,111,[113], [114], [115], [116]]). These models and techniques enable efficient resource management, predictive maintenance, and improved decision-making, thereby enhancing the functionality and sustainability of urban systems (e.g., Almalaq and Zhang, 2018; [50,[117], [118], [119], [120]]).

Worth noting is that ML, DL, CV, and NLP fundamentally intersect to address complex problems, especially in urban management and planning, where integrated models leverage cross-domain insights for enhancing urban systems (e.g., Ref. [31,55,86,109,[121], [122], [123]]). In data-driven urban planning, UDT bridges the digital and physical domains to enable real-time monitoring, assessment, and prediction of urban system behaviors, while ML, DL, CV, and NLP techniques analyze vast datasets, exemplifying their combined potential when seamlessly integrated [64]. Bibri [55] provides an in-depth analysis of the synergistic integration of ML, DL, CV, NLP, and Generative AI (GenAI) subfields, emphasizing their role in fostering collaboration and innovation in data-driven environmental planning by driving the development of AI systems that advance sustainable smart cities. While GenAI falls outside the immediate scope of this study, it is increasingly recognized as a critical enabler at the frontier of urban intelligence and decision-making, particular in the context of environmental sustainnability. Indeed, it is becoming more deeply integrated into both UDT systems (e.g.,Canzaniello et al., [124]; Huang et al., [125]; Razavi et al., [126]; Xu et al., [127]) and AIoT-driven UDT frameworks—under the emerging paradigm of Generative AI of Things (GAIoT)—to accelerate environmental outcomes in sustainable smart city development [128].

#### 4.1.4 Cyber-Physical Systems versus Cyber-Physical Systems of Systems: key characteristics, components, distinctions, and applications

The evolution of sustainable smart cities has driven significant advancements in integrating CPS into their frameworks. These systems represent a notable innovation, seamlessly connecting the digital and physical domains to optimize various urban processes. However, as urban challenges grow in complexity, the need for more interconnected and adaptive systems has given rise to CPSoS, particularly in the form of complex city platforms such as UB and UDT. Unlike CPS, CPSoS unifies multiple CPS setups into a cohesive and interconnected framework, enabling the management of diverse and large-scale objectives, including environmental sustainability and long-term urban resilience.

CPS are tightly integrated networks that connect computational algorithms (cyber) with physical processes through sensors, actuators, and control systems. These systems enable seamless interaction between digital systems and real-world environments, facilitating dynamic monitoring and control of specific urban operations. They enhance system responsiveness, automation, and efficiency through feedback loops [129], making them indispensable for enhancing the performance of urban systems. They operate with localized control and optimization, leveraging AI technologies, especially ML and DL, to focus on specific tasks within clearly defined environments [80,81]. Communication in CPS is typically limited to direct interactions between system components, utilizing a centralized or task-specific decision-making approach. For instance, a traffic light system employs sensors and algorithms to monitor real-time traffic conditions, dynamically adjusting signal timings to optimize traffic flow and reduce congestion as part of UDT. Another example is a CPS-based smart grid that adjusts energy distribution in real-time to balance demand and supply, reducing energy losses and enhancing efficiency as part of UB.

CPS integrates multiple components that collectively enhance the functionality, efficiency, and sustainability of urban systems. These components include sensing systems that collect data, robust communication networks that transmit these data, advanced AI-driven computational units that generate actionable insights, actuation systems that translate these insights into physical actions, and a software layer that orchestrates automation and scalability (e.g., Ref. [53]; Nizar et al., 2022; [81]). To maintain system integrity, these components are safeguarded by robust security and privacy measures (e.g., Ref. [[130], [131], [132]]). Table 3 illustrates these components, as distilled from these studies, alongside examples of their applications in urban and industrial systems (e.g., Ref. [25,27,28,41,56,58,133]). It emphasizes the roles and interdependencies of these components in creating cohesive and adaptive systems, highlighting their contributions to enhancing the operational functioning of urban systems.

**Table 3:** Key components of Cyber-Physical Systems and their applications in urban and industrial systems.

Component	Description	Examples in Urban and Industrial Systems
**Sensing**	Collects real-time data from the physical environment using sensors. Measures environmental parameters such as air quality, traffic flow, and energy usage to provide foundational inputs for decision-making processes.	Sensors in smart traffic systems detect congestion, air quality monitors in urban areas, or energy meters in smart grids.
**Communication**	Transfers data seamlessly between components, ensuring interoperability and enabling real-time responses. Facilitates data exchange between sensing layers, computational units, and control systems.	Communication networks in IoT-enabled systems, such as vehicle-to-infrastructure (V2I) communication or smart grid data transfer protocols.
**Computation and Control**	Processes and analyzes incoming data using algorithms to transform raw information into actionable insights. Incorporates AI-driven analytics, including ML, DL, and predictive modeling, to optimize operations and anticipate future scenarios.	AI models in smart cities analyze traffic patterns to reduce congestion, while ML systems optimize energy distribution.
**Actuation**	Converts computational insights into physical actions. Responsible for implementing real-time interventions such as adjusting infrastructure settings or controlling mechanical operations.	Adaptive traffic light systems, dynamic energy allocation in smart grids, or automated environmental monitoring systems.
**System Integration and Coordination**	Facilitates the orchestration of hardware components and computational algorithms, ensuring interoperability, scalability, and efficient deployment across diverse systems. Incorporates user interfaces for real-time monitoring and control, offering critical insights, diagnostics, and actionable feedback to enhance system performance.	Platforms managing smart grid operations, urban mobility systems, or building automation, with real-time dashboards for operators to make informed decisions.
**Security and Privacy**	Protects sensitive data and systems from cyber threats while ensuring public trust. Includes encryption, secure communication protocols, and privacy safeguards for data related to individuals or communities.	Cybersecurity frameworks for smart energy systems or privacy-preserving data analytics in urban mobility applications.

In urban and industrial systems, the seamless interaction of CPS components creates an interconnected ecosystem capable of real-time decision-making and adaptive responses. This dynamic interplay establishes a continuous feedback loop, enabling CPS to adapt to changing conditions while optimizing efficiency and functionality. CPS plays a key role in advancing sustainable smart cities by enabling real-time monitoring, coordination, and control of complex systems.

Moreover, CPS serves as the technological backbone of a wide range of AIoT-driven applications in sustainable smart city management and planning [25]. Regarding planning, the adaptive strategies supported by CPS can indirectly inform UDT for short-term predictive adjustments. For instance, UDT excels in predicting urban mobility patterns over days or weeks [134], e.g., forecasting the short-term impacts of rerouting strategies. Conversely, immediate, localized simulations for operational management, such as optimizing signaling systems in response to real-time traffic bottlenecks, are more characteristic of UB [59], leveraging CPS and AIoT for dynamic adjustments.

Furthermore, CPS enhances urban management and planning in the context of environmental sustainability, aligning short-term actions with long-term goals to address pressing challenges in resource optimization, energy efficiency, and ecological resilience [18,25]. In this context, Alexandra et al. [56] examine the transformative impact of CPS in water management and governance, emphasizing their deployment across diverse sectors and river basins. CPS, characterized as AI-enabled and automated smart systems, are reviewed for their applications in monitoring, managing, and governing hydrological systems across the water cycle in rural, urban, and coastal contexts. The research highlights the importance of integrating socio-ecological dimensions into CPS design and implementation.

Rane et al. [48] emphasize the critical role of AI and ML in transforming renewable energy strategies, with applications directly aligning with the principles and functionalities of CPS. Enhancing forecasting capabilities allows AI and ML to predict and optimize energy production and distribution, exemplifying a core aspect of CPS: the seamless integration and dynamic management of data-driven processes within physical systems. These technologies further enhance renewable energy systems through advanced analytics, predictive maintenance, and real-time decision-making in smart grids, reflecting CPS's feedback-driven control mechanisms that aim to optimize performance and minimize waste.

In the context of energy storage, AI and ML improve operational efficiency by forecasting storage demands and optimizing charge-discharge cycles, aligning with CPS's goals of resource efficiency and adaptability. The study also underscores the interplay between AI, IoT, blockchain, and edge computing in renewable energy systems. These interconnected technologies mirror CPS architectures by utilizing IoT devices for real-time data collection, edge computing for decentralized data processing, and blockchain for secure and transparent operations—all essential for maintaining system reliability and scalability [91,135,136]. This integration of advanced AI and ML techniques within renewable energy systems exemplifies CPS principles, particularly in creating tightly coupled systems where computational intelligence interacts dynamically with physical processes. The study demonstrates how such CPS frameworks can optimize energy systems while contributing to broader sustainability and environmental impact mitigation goals.

In light of the above, Sharma and Sharma [81] discuss the growing importance of CPSs as they become increasingly data-rich, enabling higher levels of autonomy and automation. While significant advancements have been made in CPS technology, there is a growing need for innovation across various applications, making foundational research crucial for keeping pace with technological developments. The study highlights how traditional CPS models are being challenged by ML and DL approaches, with AI playing a key role in analyzing data from actuators and sensors. AI is shown to enhance both system optimization and security. However, the study by Bundas et al. [137] examines the challenges inherent in integrating AI and ML into CPS and IoT technologies. While these innovations hold innovative potential, the unpredictable and often opaque behavior of AI components poses considerable barriers to their adoption. The authors propose extending an established CPS framework to incorporate unified terminology and collaborative methodologies. These advancements aim to enable designers, operators, and stakeholders to define, validate, and manage behavioral requirements for AI-driven CPS and IoT systems, thereby enhancing system understanding, reliability, and trustworthiness.

Within the framework of CPSoS, Wang et al. [87] contribute to the conceptual understanding of CPSoS by presenting a distributed IoDT architecture. This framework interconnects diverse physical systems and their digital counterparts through intra-twin and inter-twin communication, facilitating real-time synchronization, dynamic mission coordination, and data exchange. The IoDT architecture exemplifies the adaptive and interconnected nature of CPSoS by enabling efficient information aggregation and composite insights across urban domains. The study also addresses critical challenges in security and privacy within IoDT systems, proposing advanced defense mechanisms to ensure the integrity and reliability of these interconnected networks. These advancements demonstrate the foundational principles of CPSoS and their transformative potential in managing complex and dynamic urban ecosystems. Building on this study, Radanliev et al. [54] present a hierarchical cascading framework designed to enhance resilience in CPS through the integration of IoT and AI. This framework enhances the understanding of AI decision-making in multiple interconnected CPS, providing valuable insights into managing complexity and enhancing system robustness.

When multiple CPS setups are interconnected and operate in coordination, they form CPSoS—an advanced iteration of CPS. CPSoS represents a network of networked interacting and interdependent systems that collaborate to achieve broader, more complex goals beyond the capabilities of individual CPS. Bondavalli et al. [84] present an in-depth exploration of CPSoSs, highlighting their critical role in underpinning modern infrastructure across various domains, such as energy grids, transportation networks, water supply systems, and embedded technologies. They emphasize the importance of ensuring the correct operation and availability of these interconnected and independent systems as they form the foundation of contemporary societal functions. The study presents a unified conceptual framework for understanding CPSoSs, examining their defining characteristics, including time, emergence, evolution, and dynamicity. Accordingly, the authors provide a framework that enhances the description, analysis, and operational understanding of CPSoSs, offering practical guidance for their development and application in critical systems.

Papaefstathiou and Hatzopoulos [85] explore the significant potential of CPSoS, an advanced evolution of CPS and a natural extension of IoT. CPSoS merges the physical and digital worlds, enabling software applications to interact directly with real-world events through globally networked data and services, fostering innovation, and creating novel platforms. The study highlights the need for future CPSoS to handle highly coordinated, complex infrastructures characterized by multi-scale, multi-layer, multi-domain, and multi-system integration. These infrastructures will demand advanced system science and engineering approaches to support their design and development. Key topics discussed include foundational principles for heterogeneous CPSoS, the role of blockchain technologies, reconfigurable systems, advanced sensor interfaces, and human-centered design. The authors also review advanced tools and methodologies necessary for designing and implementing CPSoS, emphasizing their potential to drive innovation across various domains.

Table 4 An overview of key comparative aspects of Cyber-Physical Systems and Cyber-Physical Systems of Systems Building on the insights from Table 2, CPSoS go beyond the scope of individual CPS setups in several critical ways, reflecting their inherently more complex, large-scale, and integrated nature—essential characteristics for effectively managing interconnected systems in diverse urban contexts. While many foundational principles remain the same, CPSoS introduces additional layers of technical considerations that address the broader scale and intricate nature of such systems. These distinctions are exemplified by UB and UDT platforms, which represent advanced implementations of CPSoS in the landscape of sustainable smart cities (see, e.g., Ref. [25,32,55,60,64,86,138]; Lei et al., 2022; [25,61,74,139]). Table 5 elaborates on these differences, providing a detailed comparison that demonstrates how CPSoS tackle challenges such as interoperability, emergent behavior, and strategic alignment, ultimately enhancing functionality and resilience in complex urban systems.

**Table 4:** Presents an overview of a comparative analysis derived from the previously reviewed studies on CPS and CPSoS. This overview involves the key attributes and functionalities of these systems while positioning UB and UDT as illustrative examples of advanced CPSoS for operational management and strategic planning, respectively.

Aspects	CPS	CPSoS (e.g., UB and UDT)
Scope	Single, self-contained system	Multi-system, interconnected, large-scale
Integration	Limited to internal components	Requires interaction and coordination across multiple CPS setups
Communication	Direct, component-level	Distributed, involving cross-system data sharing
Control	Centralized or localized	Decentralized and collaborative
Objective	Task-specific (e.g., traffic optimization, energy management)	Holistic and multi-domain (e.g., sustainable urban management or planning)
Examples	Smart traffic lights, autonomous vehicles, localized energy grids	Integration of UB for real-time management and UDT for strategic planning with two or more domains

**Table 5:** Comparative analysis of technical components in cyber-physical systems and cyber-physical systems of systems.

Dimensions	CPS	CPSoS
Scale and complexity	Focuses on integrating cyber and physical components within a single system, often targeting specific, localized applications (e.g., traffic management, smart grids).	Requires managing multiple interconnected CPS setups that interact and depend on each other. Addresses scalability, emergent behavior, and system-wide optimization.
Interoperability and integration	Ensures seamless interaction between the cyber and physical components within a single domain.	Harmonizes communication protocols, data formats, and control mechanisms across heterogeneous CPS setups operating in different urban domains.
Data management and coordination	Focuses on real-time data processing and analytics for a specific application.	Manages vast volumes of data across multiple CPS setups, ensuring efficient aggregation, distribution, and analysis while avoiding redundancies.
Feedback loops and decision-making	Implements localized feedback loops to monitor and control system behavior.	Requires sophisticated bidirectional feedback mechanisms integrating localized responses with system-wide strategic objectives.
System resilience and robustness	Ensures resilience at the system level, focusing on fault tolerance and recovery within a single application.	Addresses resilience at a higher level, ensuring disruptions in one CPS do not propagate across the system of systems. Distributed control mechanisms are key.
Emergent behavior and predictability	Relatively predictable due to the bounded scope of interactions within the system.	Can exhibit emergent behavior due to interactions among multiple CPS setups, necessitating management of complex dynamics.
Security and privacy	Secures individual system components and their communication channels.	Faces amplified security challenges and privacy concerns due to interconnected systems, increasing vulnerability to cascading failures and cyberattacks.
Real-time and strategic alignment	Primarily emphasizes real-time operational performance.	Balances real-time responsiveness with strategic objectives, requiring coordination between operational analytics (e.g., UB) and predictive simulations (e.g., UDT).
Governance and control	Often managed by a single entity with centralized governance.	Requires decentralized governance to align goals, share data, and ensure equitable resource allocation among multiple stakeholders and jurisdictions.
Energy efficiency and sustainability	Focuses on optimizing the energy efficiency of individual systems.	Requires systemic strategies to optimize energy usage across interconnected CPS setups for sustainability while maintaining overall performance.

While CPSoS builds on the technical foundations of CPS, it introduces additional challenges related to scale, complexity, and interconnectivity. These considerations necessitate advanced approaches to design, implementation, and governance, making CPSoS a distinct and more intricate domain in the broader spectrum of CPS [84,85]

Operating at a larger scale, CPSoS forms a cohesive ecosystem capable of managing and planning across diverse domains or a single domain throughout the city. For example, within a CPSoS framework, multiple CPS-based smart grids are coordinated to dynamically optimize energy distribution across city districts while seamlessly integrating renewable energy sources and storage systems to enhance resilience and sustainability. Unlike CPS, CPSoS focuses on system-level coordination and also involves multiple domains (mobility, energy, water, waste, materials, environment, etc.)—working collaboratively to optimize complex processes as part of large-scale AIoT systems such as UB and UDT. These systems emphasize interoperability, scalability, and adaptability across these domains. Communication and control in CPSoS are distributed, requiring extensive cross-system data exchange and dynamic collaboration to address multifaceted challenges in urban systems. This process is reflected in UB and UDT in the context of smart cities (e.g., Ref. [25,59,73,74]) and sustainable smart cities [55,60,61,86].

Within the framework of UDT, for instance, Huang et al. [86] introduce a UDT platform that exemplifies the principles of CPSoS by highlighting the importance of system-level integration, interconnected operations, and adaptive planning in addressing the multifaceted challenges of environmental sustainability in smart cities. While not directly referring to CPSoS, the platform demonstrates how localized computational systems and physical processes—spanning mobility patterns, vehicle movements, energy monitoring, waste management, material usage, and biodiversity tracking—can function cohesively within a unified framework. This integration underscores the potential of interconnected CPS setups to operate collaboratively, providing a comprehensive and adaptive approach to urban sustainability. Coordinating diverse urban domains enables the UDT platform to ensure synchronized planning across interconnected systems. Through real-time data integration and simulation, the UDT dynamically adapts to evolving urban conditions. Moreover, the UDT platform synthesizes data across domains to generate strategic insights, such as estimating flow data in new locations, forecasting the evolution of flow data, and informing infrastructure development that aligns with environmental sustainability objectives. This multi-domain approach highlights the crucial role of CPSoS in achieving comprehensive and sustainable urban planning.

#### 4.1.5 The dynamic interplay of artificial intelligence of the Internet of Everything and Cyber-Physical Systems of Systems

The convergence of emerging technological paradigms is transforming how urban systems are managed, optimized, and designed, with a distinct focus on environmental sustainability. Among these paradigms, **CPSoS** and **AIoE** emerge as synergistic and groundbreaking integrated frameworks. AIoE builds upon the foundational connectivity of IoT by encompassing a broader ecosystem of interconnected devices, systems, processes, people, and environments. Through advanced AI-driven analytics, AIoE enables improved decision-making, enhanced pattern recognition, streamlined process automation, and optimized system performance across this interconnected ecosystem. This evolution fosters smarter interactions and integrations within urban systems, promoting holistic and adaptive strategies for tackling complex urban challenges. Together, CPSoS and AIoE constitute the backbone of advanced urban ecosystems, enhancing capabilities such as real-time monitoring, predictive insights, and cross-domain coordination. The interplay between these paradigms creates new avenues for addressing the intricate challenges of environmental sustainability and urban resilience. Their complementary roles and dynamic interactions highlight the potential of CPSoS and AIoE to drive technological innovation and advance data-driven approaches to management and planning for sustainable and resilient urban futures.

A CPS framework incorporates a wide array of technologies, including AI, ML, DL, IoT, big data analytics, IIoT, blockchain, edge and cloud computing, and 5G/6G networks, to create interconnected and resilient systems [81,91,135]. This integration transforms AIoT-driven insights into actionable measures, empowering sustainable smart cities to adapt dynamically to real-time demands while aligning with long-term sustainability goals [25,38]. By merging AI's analytical and computational capabilities with the expansive connectivity of AIoE and the operational precision of CPSoS [85,140,141], these frameworks collectively enable advanced, real-time decision-making, holistic system optimization, and enhanced prediction. Their interplay enhances the functionality of urban systems while leveraging synergies that address the interconnected challenges of environmental sustainability, adaptive urban management, and strategic planning. This convergence underscores the transformative capabilities of CPSoS and AIoE paradigms in driving efficiency, resilience, and sustainability within urban ecosystems.

As a critical enabler of sustainable smart cities, IoT serves as the foundational infrastructure, deploying a network of sensors and devices to generate extensive real-time data streams that drive sustainable development (e.g., Ref. [142,143]). These data streams are then processed and analyzed by **AI systems** using **ML** and **DL**, enabling informed decision-making to support the development of sustainable smart city infrastructures [6,35]. Enhancing this ecosystem, IoE strengthens the integration of urban systems by fostering intelligent, interconnected ecosystems that can adapt and respond dynamically to the complexities of evolving urban challenges. This interconnected framework is supported by the critical role and significance of smart sensors and actuators in IoE-enabled smart city infrastructure, as highlighted by Singh et al. [144], whose contributions to real-time data collection and intelligent responsiveness are improving the performance and sustainability of urban environments. Smart sensors and actuators enable proactive management and optimization by gathering data from monitoring public infrastructure. The study highlights their critical applications and the opportunities they provide in shaping smarter, safer, and more sustainable urban environments.

Souza et al. [145] emphasize the need for collaborative and efficient computing architectures as IoT technologies continue to transform urban areas into smart cities. Extending beyond traditional machine-to-machine interactions, the Internet of Everything (IoE) incorporates human-to-human and human-machine collaboration. The study evaluates four key IoE computing architectures: Edge Computing, Cloud Computing, Blockchain/Web Services, and Fog Computing. Using the 3C collaboration model—communication, cooperation, and coordination—the authors assess the capabilities and limitations of these architectures. Their findings emphasize the critical roles of Edge Computing, which enables real-time, low-latency processing at data sources, and Cloud Computing, which offers scalable resources for diverse workloads. These architectures enhance collaborative coordination by improving efficiency and network functionality. Effective resource allocation and network configuration are identified as critical for cohesive IoE ecosystems. The study provides valuable insights for selecting the optimal computing frameworks in IoE environments. Applying the 3C collaboration model provides a practical approach to enhancing collaboration in smart city systems.

Given the above, IoE facilitates seamless communication and data sharing among all connected entities, creating an interconnected smart city infrastructure that links devices, systems, and people. Building on this foundation, AIoE integrates autonomous intelligence into these interconnected systems, enabling innovative, self-learning technologies that not only share and analyze data but also make and execute decisions independently. Solanki and Singh [141] explore how intelligent systems and IoE are driving the transformation of smart cities, making them more efficient, interconnected, and responsive to citizen needs. The authors provide a detailed examination of the potential and challenges associated with IoE-based smart cities powered by advancements in AI and ML. Key topics include design considerations for the physical layer that supports smart city infrastructure, enabling technologies for intelligent systems within smart computing environments, the role of smart sensors and actuators, and their associated challenges and future trends. Additionally, the study addresses the applications, enabling technologies, and emerging challenges of IoE while highlighting future directions for smart city development.

Lawless et al. [146] delve into the foundations, metrics, and applications of AIoE, examining its impact on devices, systems, and broader ecosystems. The work explores critical questions about whether IoE systems and devices should interact exclusively with one another, with humans, or with both, and considers how these interactions influence society at large. It examines the pivotal role of IoE in shaping future ecosystems, highlighting its impact on sensing, perception, cognition, and behavior within the context of intelligent and autonomous systems. The study also addresses practical, theoretical, and research challenges, focusing on the implications of IoE systems as they begin to reason, communicate, and act autonomously. A particular emphasis is placed on their potential to function independently or interdependently, raising critical questions about the societal and systemic impacts of autonomous and interconnected “things.”

Overall, the key elements of AIoE in sustainable smart cities include real-time data collection and analysis, intelligent decision-making, predictive and adaptive capabilities, system integration and interoperability, scalability and resilience, as well as sustainability-focused optimization. These elements are brought together through CPS to enable integrated dynamic management and adaptive planning processes.

Within the framework of CPS, Haldorai [52] examines the integration of AI, IoT, and CPS, highlighting their potential to drive the next generation of smart technological advancements. The study examines the components of IoT-CPS and explores the critical role of AI in enhancing their functionality. It also addresses challenges associated with managing the vast amounts of data generated in these systems, particularly the limitations of current processing capabilities. It emphasizes the need for more reliable AI systems to address these challenges and advocates for their integration into real-world IoT-CPS applications to unlock their full potential. Building on this, Song et al. [147] explore the integration of AI into CPS, highlighting the importance of benchmarking and performance evaluation. While AI-enabled CPS has seen widespread adoption across industries, the study highlights gaps, including a lack of publicly available benchmarks and a limited understanding of their performance and reliability across diverse domains. To address these gaps, the authors develop a public benchmark utilizing advanced Deep Reinforcement Learning (DRL) methods. Systematic evaluations comparing AI-enabled CPS with traditional systems reveal critical challenges, such as the need for enhanced testing techniques and deeper exploration of hybrid CPS to improve performance and reliability.

Within the broader framework of CPS, Masserov and Masserov [140] explore the innovative potential of integrating IoT with AI. The authors envision a future characterized by the "Internet of Everything on a Smart Cyber-Physical Earth," positioning IoT combined with AI as a pivotal driver of the next technological revolution. They emphasize that the primary challenge lies in processing vast amounts of data with the limited computing power currently available, a hurdle that advancements in AI and data science aim to address. They highlight the multifaceted benefits of integrating IoT and AI, extending beyond cost savings and efficiency to enhancing human lives and meeting the evolving needs of society. They argue that the success of this integration depends not only on technological advancements but also on how these systems are perceived—whether as beneficial tools, unnecessary burdens, or potential threats.

This vision aligns with the concept of CPSoS within the broader paradigm of a Smart Cyber-Physical City. In this advanced framework, interconnected and intelligent systems collaborate to optimize processes across diverse urban and industrial domains, leveraging the innovative potential of AIoT to create adaptive and resilient ecosystems. This study highlights their capacity to address complex challenges by integrating AI and IoT within the CPSoS and Smart Cyber-Physical City frameworks, advancing environmental sustainability and fostering innovation on an unprecedented scale. Within the CPSoS framework, AIoE plays a crucial role by enabling seamless communication and integration between the UB and UDT ecosystems, thereby forming a unified framework for intelligent, real-time monitoring, operational management, predictive planning, and data-driven strategic decision-making. Through the computational power of AI and the data-generation capacity of IoT, this connectivity enables the seamless integration of key urban domains—including energy, transportation, waste, and environmental monitoring—while fostering a dynamic and synergistic interplay between UB and UDT functionalities. This ensures synchronized responses to immediate challenges and alignment with long-term objectives, further solidifying the role of CPSoS in driving environmentally sustainable smart city development [25].

### 4.2 Architectural and comparative insights into artificial intelligence of things-driven ecosystems

As urban systems grapple with escalating sustainability challenges, AIoT-driven systems for urban management and planning have emerged as pivotal tools within CPSoS frameworks. These advanced systems harness the powerful convergence of AI and IoT technologies to enhance real-time operational functionalities and strategic planning capabilities, respectively, equipping cities to navigate the multifaceted complexities of urban life effectively. The architectural and comparative insights detailed next establish a foundational understanding of the distinctions and synergies between UB and UDT, emphasizing their contributions to advancing urban management and planning within AIoT-driven ecosystems.

#### 4.2.1 Architectural layers and integration frameworks of artificial intelligence of things grounded in the data science cycle

AIoT architectures, grounded in the iterative principles of the data science cycle, are designed to optimize and advance urban systems. While their design and implementation may vary across domains, these architectures share a core objective: the intelligent collection, analysis, and application of data to enhance the functionalities of AIoT-driven systems such as UB and UDT. The studies summarized in Table 5 showcase a diverse range of architectural layers, computational processes, and integration frameworks, reflecting the dynamic evolution of AIoT in the rapidly evolving landscape of sustainable smart cities. Notably, some architectural layers overlap with computational processes, which are referred to as the pillars of AIoT systems. Table 6 presents a comparative overview of AIoT architectures, emphasizing their key differences and shared attributes. It highlights their unique contributions and significant impacts on advancing sustainable urban ecosystems.

**Table 6:** A comparative overview of Artificial Intelligence of Things architectures and their characteristics.

Architectural Layers	Key Computational Processes	Integration Frameworks	Contributions/Insights	Citation
Tri-tier structure comprising edge, fog, and cloud computing. Four cognitive processes: Perceiving, learning, reasoning, and behaving.	Perception-Action Cycle in intelligent systems to enable adaptive and autonomous functionality.	Distributed architecture with centralized cloud topology.	Emphasizing distributed edge and fog nodes, along with centralized cloud resources, for enhanced AIoT system performance.	Zhang and Tao [111]
Five cognitive processes: sensing, perception, learning, visualization, and acting.	Transforming sensory data into actionable insights using ML and DL and visualization for real-time insights.	Focus on interrelated processes enhancing real-time decision-making in urban domains.	Introducing a structured computational process tailored for optimizing urban systems.	Bibri et al. [22]
Six cognitive processes: adding evaluation between learning and visualization.	Introducing evaluation metrics and iterative feedback to assess outcomes to refine learning and reasoning before proceeding to visualization and action.	Including feedback mechanisms for model validation and reliability.	Enhancing model performance and ensuring meaningful data-driven insights.	Bibri et al. [93]
Three-step system: IoT data analytics systems, APIs, and AI/ML systems.	Predictive modeling, scenario forecasting.	API-enabled interoperability across devices, software, and platform levels.	Highlighting APIs' role in ensuring seamless data integration and communication.	Samadi [148]
Three layers: sensors/devices/energy, communication and networking, and applications.	Computational frameworks for data handling across edge, fog, and cloud layers.	Integrating AIoT with 5G/6G networks and emerging communication technologies.	Exploring hardware and connectivity innovations for scalable and efficient AIoT implementations.	Seng et al. [50]
Decentralization, edge computing, and hardware accelerators.	Model compression, quantization, energy-efficient algorithms, latency reduction.	Emphasizing scalable architectures through decentralized systems and real-time decision-making.	Introducing optimization strategies such as model compression and energy-efficient algorithms to overcome hardware limitations.	Yuan [149]

The comparative analysis highlights the diverse approaches to AIoT architectures, with each study presenting distinct layers or processes designed to address specific urban challenges while demonstrating adaptability across varied applications. Common elements among these architectures include foundational reliance on sensors, networking, and computing layers. However, notable differences arise in their computational mechanisms and approaches to advanced connectivity. These distinctions highlight the ongoing innovation in aligning AIoT architectures with the iterative principles of the data science cycle, addressing the unique urban contexts and objectives. Moreover, these frameworks lay a foundation for future advancements in AIoT by emphasizing the importance of scalability, seamless interoperability, and domain-specific customization to meet the evolving demands of sustainable smart cities. Together, these architectures illustrate the vast potential of AIoT to drive transformative changes in urban system functionalities, enabling more integrated, adaptive, and scalable solutions for sustainable urban development [6,13,25,29,35,39,42,47,48].

Notably, recent advancements in edge computing are reshaping the IoT and AIoT ecosystems, addressing challenges related to data processing, scalability, and latency. The early studies by Yang et al. [150] and Yang et al. [151] explore the *innovative capabilities* of edge computing technologies, highlighting innovative solutions to overcome limitations and enhance the efficiency of IoT infrastructure, particularly for AI-powered applications. Yang et al. [150] focus on the increasing reliance on edge computing as an alternative to cloud-device architectures, which struggle to handle the vast data volumes generated by IoT devices. They emphasize that edge computing, by bringing data processing and storage closer to the network edge, offers key benefits, including reduced latency, improved scalability, and increased energy efficiency. Yang et al. [151] introduce an innovative edge-based IoT platform designed specifically for AI applications. The study addresses challenges related to the deployment and management of edge data centers. This platform provides a unified interface for resource optimization and incremental deployment. The study's simulations and real-world deployments demonstrate significant performance improvements, validating the platform's effectiveness in optimizing AI-driven IoT systems. These two studies underscore the critical role of edge computing in advancing AIoT ecosystems.

### 4.3 Comparative analysis of Urban Brain and Urban Digital Twin architectures

Urban systems are undergoing a profound transformation fueled by advanced technological paradigms, particularly in the realms of UB and UDT. These systems serve as critical elements of the broader CPSoS framework, leveraging AIoT technologies to effectively merge real-time operational insights with forward-looking strategic planning. UB is tailored to enhance urban operations through real-time monitoring, advanced analytics, and immediate decision-making, whereas UDT emphasizes predictive modeling, scenario analysis, and long-term strategic foresight. Together, UB and UDT architectures address the multifaceted challenges of urban management and planning, fostering efficient and resilient urban ecosystems.

According to numerous foundational and practical studies, UB platforms harness advanced AI computational and analytical capabilities to extract actionable insights from vast and heterogeneous urban data generated via IoT sensors and devices and other sources to transform urban management (e.g., Ref. [59,60,62,74,75,93,94,95]) through domain-specific stages such as cognition, optimization, decision-making, prediction, and intervention [76,77]. Conversely, UDT platforms generate dynamic, high-fidelity digital representations of physical urban environments, enhancing urban planning processes. Leveraging real-time data from IoT sensors, GIS mapping, satellite imagery, and other urban repositories, UDT integrates AI-driven modeling, simulation, and predictive analytics to enable scenario-based planning and strategic goals, supporting informed decision-making by allowing planners to visualize potential outcomes, test policy interventions, and optimize urban systems (e.g., Ref. [55,63,64,67,73,127,139,152]).

Based on the relevant insights distilled from the studies above, Table 7 presents a comprehensive comparative analysis of the architectural layers of UB and UDT systems in terms of their underlying technical components. Derived and adapted from a recent study by Bibri [153], it highlights the structural and functional attributes of these approaches, emphasizing their shared objectives in advancing urban development and their distinct roles in real-time operational management (UB) and strategic predictive planning (UDT). Together, UB and UDT architectures exemplify the synergistic and complementary functionalities that are crucial within CPSoS frameworks, enabling the alignment of immediate responses with long-term objectives. This integrated approach facilitates the development of sustainable smart city ecosystems, addressing current urban complexities while preparing for future challenges.

**Table 7:** Comparative analysis of architectural layers of Urban Brain and Urban Digital Twin.

Technical Components of UB and UDT	Description	Differences
IoT layer (UB)	Distributed across urban platforms and systems to gather real-time data through connected sensors and devices for monitoring and responsiveness.	UB focuses on operational real-time monitoring and action, while UDT uses IoT data and other forms of data for detailed modeling and predictive simulations.
Big data layer (UB)	Collects, stores, and processes extensive raw data to extract actionable insights for decision-making and management.	UB emphasizes large-scale operational insights, whereas UDT integrates diverse data types for dynamic simulations and scenario analysis.
Cloud computing layer (UB)	A centralized system that manages data with high computational power, enabling the efficient integration and coordination of urban processes.	UB utilizes centralized management for real-time urban operations; UDT leverages cloud services for modeling, analytics, and scalability.
Edge computing layer (UB)	Processes data at the source with AI-enabled devices, reducing latency and enabling immediate decision-making.	UB relies heavily on edge computing for real-time data processing and instantaneous urban responses, while UDT, though primarily dependent on centralized systems, incorporates edge computing for faster synchronization and simulation updates in dynamic environments.
AI layer (UB)	Applies ML, DL, CV, and GenAI algorithms for real-time data analysis, data generation, adaptive learning, and operational optimization.	UB emphasizes pattern recognition and urban process optimization; UDT focuses on predictive modeling and long-term scenario planning.
Internet neural network layer (UB)	Interconnects urban components, supporting collaborative interactions among humans, things, and systems.	UB enhances system-level collaboration; UDT focuses on integrating feedback mechanisms for model refinement.
Data acquisition and integration layer (UDT)	Collects and integrates data from IoT devices, GIS, satellite imagery, and other sources for a holistic urban representation.	Exclusive to UDT for comprehensive and up-to-date urban data collection.
Modeling and simulation engine (UDT)	Core engine enabling dynamic simulations, scenario planning, and predictive modeling of urban environments.	Exclusive to UDT for scenario-based strategic foresight and planning.
AI and analytics module (UDT)	Processes real-time data for insights using ML, DL, CV, NLP, and GenAI algorithms for predictive analysis, anomaly detection, optimization, visual monitoring, textual data interpretation, and synthetic data generation for enhanced urban system simulations.	A **comparable** use of AI exists in both UB and UDT, but UDT employs AI primarily for predictive and scenario modeling.
Visualization and user interfaces (UDT)	Provides stakeholders with real-time visualization tools to explore urban dynamics and scenarios and enable data-driven decision-making.	Focused on user-centric exploration and planning tools, distinct from UB's operational visualization for urban monitoring.
Communication protocols (UDT)	Facilitates seamless data exchange and interoperability between UDT components.	UDT relies on communication protocols tailored for internal data flow between digital and physical components, while UB emphasizes real-time system communication to support operational tasks.
Security infrastructure (UDT)	Protects sensitive urban data, ensuring privacy, integrity, and resistance to cyber threats.	Essential for both UB and UDT, but UDT places additional emphasis on securing simulation and predictive data.
Semantic knowledge representation (UDT)	Enhances understanding of urban features and relationships to support informed decision-making.	UDT-specific for improved interpretability and actionable insights in simulations and **scenario analyses.**
Feedback mechanism (UDT)	Continuously refining models using real-world data, UDT enhances its predictive capabilities over time, ensuring its simulations remain accurate and aligned with evolving urban dynamics.	Core to UDT, feedback mechanisms enable ongoing model refinement and synchronization with real-world systems, whereas UB focuses primarily on real-time operational adjustments and lacks explicit iterative feedback loops.
Scalability and flexibility (UDT)	Adapts to expanding urban environments and emerging technologies, addressing new challenges.	UDT emphasizes long-term scalability and flexibility, while UB focuses on immediate scalability to meet operational demands.

By harmonizing the complementary capabilities of UB and UDT, these data-driven management and planning systems create a robust framework for tackling the multifaceted challenges of sustainable urban development. They achieve this by enhancing resource efficiency, promoting environmental sustainability, and strengthening urban resilience, which is the focus of the next subsection.

### 4.4 The role of Urban Digital Twin and Urban Brain in advancing environmental sustainability through artificial intelligence and artificial intelligence of things

The integration of AI and AIoT into UB and UDT systems has emerged as a transformative force in advancing environmental sustainability in smart cities. These AIoT-driven systems facilitate a paradigm shift from reactive to proactive urban management and planning, respectively, enabling cities to address and anticipate environmental challenges more effectively. Together, they establish a comprehensive framework that aligns short-term urban operations with long-term urban strategies. This synthesis, presented in both textual and tabulated formats, underscores the specific management roles of UB and the distinct planning functions of UDT, illustrating their unique yet complementary contributions to advancing the environmental objectives of sustainable development.

In a recent comprehensive systematic review, Bibri et al. [64] explore the innovative potential of integrating AI, AIoT, and UDT systems within the framework of sustainable smart cities. The authors focus on how these technologies work collaboratively to reshape data-driven planning strategies, emphasizing their critical role in achieving environmental sustainability goals. The study reveals untapped synergies and complex dynamics among these interconnected technologies and domains, which drive innovation in sustainable urban development. A key focus of the study is the integration of AI and AIoT processes with UDT functionalities, which amplifies the predictive and strategic capabilities of urban planning processes, providing a roadmap for fostering environmentally conscious and resilient urban systems. The insights presented in Table 8, which detail UDT's planning functions for environmental sustainability, are derived from this comprehensive review and hence substantiated by the theoretical frameworks and practical implementations underpinning this work. The outcome underscores the augmented UDT's capacity to address complex environmental challenges through innovative approaches to data-driven urban planning.

**Table 8:** Planning functions of Urban Digital Twin in relation to environmental sustainability.

UDT domains	Description	Relevance to Environmental Sustainability
**Resource allocation**	Optimizes the distribution and utilization of natural and urban resources based on real-time and predictive data insights.	Reduces waste, ensures equitable resource distribution, and prevents overexploitation of finite resources.
**Infrastructure development**	Simulates urban infrastructure projects to evaluate their long-term environmental impacts.	Promotes green infrastructure, reduces carbon footprints, and supports resilient urban growth.
**Energy forecasting**	Utilizes data-driven modeling to forecast energy demand and supply dynamics across urban areas.	Encourages energy efficiency, reduces dependency on non-renewable resources, and integrates renewable energy solutions.
**Renewable energy deployment**	Models the optimal placement and scaling of renewable energy sources such as solar panels and wind turbines.	Accelerates the transition to clean energy and reduces greenhouse gas emissions.
Zero-energy buildings	Models energy consumption, renewable energy generation, and efficiency scenarios to enable precise forecasting, adaptive energy strategies, and effective decision-making throughout the lifecycle of ZEB projects.	Supports the design and operation of ZEBs to reduce carbon emissions, enhance energy efficiency, and promote the use of renewable energy sources.
**Climate scenario analysis**	Simulates various climate scenarios to evaluate the potential impacts of climate change on urban systems.	Aids in developing proactive strategies for climate mitigation and adaptation.
**Biodiversity preservation**	Monitors and plans for the conservation of urban green spaces and natural habitats.	Maintains ecological balance, supports urban biodiversity, and mitigates the effects of land-use alterations.
**Pollution Monitoring and Mitigation**	Tracks air and water pollution levels and suggests actionable strategies for remediation.	Improves environmental health and reduces adverse impacts on public health and ecosystems.
**Waste management strategies**	Analyzes urban waste streams and models efficient waste collection, recycling, and disposal systems.	Promotes circular economy principles and minimizes waste-related pollution.
**Water resource management**	Models urban water cycles, including demand forecasting, flood risk assessment, and stormwater management.	Ensures sustainable water usage, reduces flood risks, and supports climate resilience.
**Transportation and Mobility Planning**	Simulates traffic flow and models public transportation systems to minimize emissions and enhance efficiency.	Reduces greenhouse gas emissions, promotes low-carbon transportation options, and enhances sustainable urban mobility.
**Disaster Risk Reduction and Resilience Planning**	Assesses vulnerabilities and models the impacts of natural disasters, such as floods, storms, and heatwaves.	Enhances preparedness, improves disaster response capabilities, and strengthens urban resilience to climate-related risks.
**Carbon Accounting and Emission Reduction**	Tracks and models urban carbon emissions, identifying key sources and evaluating the impact of mitigation policies.	Supports the achievement of net-zero goals and the development of low-carbon cities.

Table 8 provides a comprehensive overview of UDT's planning functions, illustrating its critical role in addressing environmental sustainability and climate change challenges. Each function contributes to creating resilient, adaptive, and sustainable urban systems.

Furthermore, Zarrabi and Doost Mohammadian [67] examine the integration of DT, IoT, and AI in advancing urban intelligence and decision-making. Their study highlights how these technologies collectively enhance sustainability, resilience, and governance in smart cities. Through a comprehensive review of literature, case studies, and theoretical frameworks, the authors examine both the benefits and challenges of implementing AIoT-driven urban intelligence systems. The fusion of DT, IoT, and AI enables real-time data collection and analysis, facilitating informed and proactive urban management and strategic urban planning. AI-driven predictive analytics optimize resource allocation, improve service efficiency, and enhance infrastructure maintenance, while IoT-enabled monitoring systems support environmental tracking, traffic management, and energy optimization. The study also highlights the role of AIoT-driven UDT in fostering digital inclusion and civic engagement by enabling participatory platforms and co-creation workshops that empower citizens to shape urban planning and ensure smart city initiatives align with community needs and priorities.

In addition, numerous studies provide domain-specific insights into the role of AI- or AIoT-driven UDT in addressing challenges and enabling innovative solutions across various facets of environmental sustainability. They include the contributions of UDT to optimizing transportation systems [73,134,154], enhancing energy management through renewable energy integration and efficiency strategies [70,155,[156], [157]] accelerating sustainability in positive energy districts [158,159], improving water infrastructure management [160], and supporting disaster response with dynamic modeling and decision-making frameworks [161]. UDT enhances the adaptability and resilience of urban systems by bridging the gap between real-time infrastructure management and long-term development strategies, fostering a data-driven approach to urban planning [64].

In light of the above, UDT plays a more significant role in environmental sustainability compared to UB, primarily due to its ability to create a detailed dynamic replica of a city's physical, spatial, and functional systems. This capability enables UDT to integrate complex interdependencies and multimodal dynamics across urban infrastructure and domains, organizational structures, and natural environments, supporting a more comprehensive and adaptive approach to urban planning and infrastructure management. This is achieved either through advanced approaches to predictive modeling and process optimization [32,63,64,66,68,158,159,162] or through conventional modeling and simulation techniques for analyzing established urban systems and long-term scenarios [72,139,[163], [164], [165], [166]] for data-driven insights or scenario-based decision making, respectively. Consequently, UDT's capacity to capture and simulate interconnected urban systems positions it as a more impactful tool for advancing environmental sustainability in urban ecosystems. In contrast, UB is primarily focused on real-time urban management and operational adjustments. This distinction is evident from the applied domains of UB and its functions (Table 9), as documented by numerous studies in the field of urban management [[59], [60], [61], [62],^66^,^74^,^75^,^77^,^93^], which reveal its limited focus on addressing broader environmental strategies.

**Table 9:** Management functions of Urban Brain in relation to environmental sustainability.

UB Domains	Description	Relevance to Environmental Sustainability
**Traffic and Transportation Management**	UB monitors and analyzes real-time traffic data to optimize traffic flow, reduce congestion, and enhance public transportation systems	Reduces vehicle emissions and fuel consumption by improving traffic flow and promoting sustainable mobility options.
**Energy Optimization**	Supports the efficient use of energy resources by monitoring consumption patterns and optimizing energy distribution in urban areas.	Enhances energy efficiency, reduces energy waste, and promotes the integration of renewable energy.
**Air Quality Monitoring**	Tracks pollution levels and provides actionable insights to improve air quality and reduce environmental hazards.	Aids in mitigating air pollution and supports clean air initiatives, contributing to healthier urban environments.
**Water Resource Management**	Facilitates real-time monitoring and optimization of water distribution systems, addressing issues such as leakage detection and efficient water usage.	Conserves water resources by preventing wastage and optimizing distribution for sustainable urban living.
**Waste Management**	Enhances urban waste collection and recycling processes through data-driven insights and real-time monitoring of waste disposal systems.	Promotes circular economy practices by improving recycling rates and reducing landfill waste.
**Public Safety and Emergency Response**	Integrates data from surveillance systems and other sources to improve city-wide safety and enable rapid responses to emergencies, including natural disasters such as floods.	Protects urban populations and reduces environmental damage by ensuring timely responses to hazards and natural disasters.

Additionally, several studies offer domain-specific insights into the role of UB in promoting environmental sustainability through data-driven technologies. For example, Kierans et al. [60] propose the "Smart City Brain" concept, highlighting UB's potential to support circular economy practices by collecting, analyzing, and sharing data to optimize resource allocation, minimize waste, and foster collaboration among stakeholders. This platform aims to advance the SDGs by integrating technological connectivity with material product data to enhance circularity. In the transportation domain, Wang et al. [62] demonstrate UB's capability in real-time emissions monitoring using operational data-driven systems. Their study establishes an intelligent platform for visualizing on-road vehicle emissions and traffic states, enabling the identification of emission hotspots and the optimization of traffic control policies. These findings underscore UB's ability to bridge gaps between traffic emissions, air quality, and human exposure through high-performance computing and visualization systems. Liu et al. [61] expand UB's contributions to broader domains, showcasing its role in driving innovation across transportation, environmental protection, and smart energy systems.

Notably, according to Cugurullo and Xu [59], the predictive scope of UB encompasses three key dimensions: environmental risk management, traffic management, and public security, thereby enabling anticipatory governance for proactive urban management. In environmental risk management, UB integrates data from environmental sensors and weather information centers to predict the impacts of extreme weather, such as urban flooding. It identifies vulnerable areas, optimizes traffic routes to prevent accidents, and informs proactive infrastructure improvements, such as reinforcing riverbanks or enhancing drainage systems. In the domain of traffic management, it utilizes CCTV cameras, traffic sensors, and predictive analytics to forecast traffic jams, accident locations, and accident severity. It prioritizes responses by determining the gravity of predicted accidents and dispatching emergency services accordingly. These predictions differ from those of UDT, which primarily focuses on long-term scenario modeling and simulation rather than the immediate, actionable insights provided by UB for real-time operational adjustments.

## 5 A pioneering foundational framework for integrating Urban Brain and Urban Digital Twin: leveraging their synergistic and complementary capabilities for advancing environmental sustainability

The proposed framework integrates UB's real-time operational management functions with UDT's strategic predictive planning functions of UDT into a unified AIoT-driven CPSoS architecture. This integration addresses the interconnected challenges of environmental sustainability by leveraging the synergistic and complementary capabilities of UB and UDT to foster seamless collaboration and deliver dynamic management and adaptive planning solutions.

### 5.1 Key components, processes, and interconnections

**Fig. 4:**
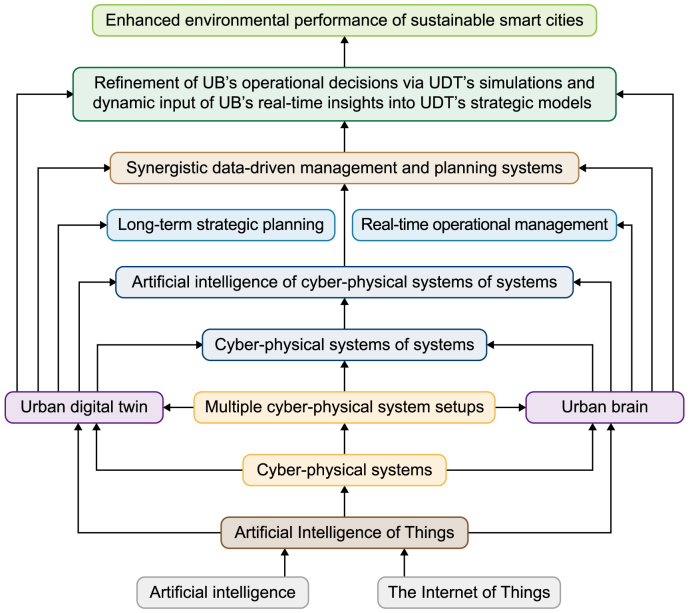
A pioneering foundational framework for integrating the functions of Urban Brain and Urban Digital Twin to enhance the environmental performance of sustainable smart cities.

The foundational framework is visually represented through its components, processes, and interconnections (Fig. 4), providing a comprehensive overview of its structure. UB and UDT represent multifaceted CPS setups, each encompassing a range of domains in urban environments. UB, as a set of real-time CPS setups, focuses on real-time analytics and operational tasks across specific domains. UDT, as a set of strategic CPS setups, extends its focus to predictive modeling and scenario-based planning across specific domains. Together, they operate as interconnected platforms within the CPSoS framework, harnessing AIoT technologies to enhance environmental sustainability and climate resilience in urban environments. The AIoT ecosystem integrates IoT's data acquisition and connectivity capabilities with AI's computational and analytical power, enabling real-time processing, predictive analytics, and intelligent decision-making within this framework. The overarching CPSoS architecture unifies UB and UDT by facilitating bidirectional communication between their physical and digital components, ensuring continuous feedback and adaptation.

The processes within the proposed framework are equally important. Data collection and processing form the foundation, where IoT sensors and devices stream real-time data to UB for operational decisions and to UDT for simulation-based predictive modeling. UB applies real-time analytics to optimize urban operations, while UDT employs predictive modeling to generate scenarios and forecast future conditions, guiding strategic planning. Decision-making and implementation are enhanced through actionable insights generated by the unified framework, enabling sustainable smart cities to respond dynamically to urban challenges while maintaining strategic alignment with overarching sustainability goals. AI and AIoT seamlessly support these interconnected processes within the proposed CPSoS framework, where their distinct yet interdependent roles enhance the functionality and effectiveness of UB and UDT, enabling dynamic urban management and adaptive planning for sustainable smart cities (Table 10).

**Table 10:** Roles and functions of Artificial Intelligence and Artificial Intelligence of Things and their synergistic integration in the foundational framework.

Aspects	Roles and functions of artificial intelligence	Examples of applications
Data analysis and interpretation	AI employs advanced algorithms, such as ML and DL, to process vast and diverse datasets generated by IoT devices, urban sensors, and other data sources. These capabilities enable pattern recognition, anomaly detection, and the prediction of future scenarios.	Recognizing traffic patterns and detecting energy inefficiencies.
Predictive modeling and simulation	AI powers UDT's predictive capabilities by generating simulations and scenario analyses to forecast the impact of policies, infrastructure developments, and environmental changes. This function supports strategic planning and long-term decision-making.	Evaluating urban growth scenarios or predicting climate adaptation impacts.
Real-time decision-making	AI processes real-time data streams in UB to provide actionable insights, enabling immediate responses to challenges such as traffic congestion, energy optimization, and environmental monitoring.	Automated traffic signal adjustments; real-time air quality alerts.
Optimization	AI ensures the cohesive and sustainable functioning of urban systems by optimizing resource allocation, energy efficiency, and operational processes.	Optimizing energy grid performance and smart waste collection systems.
Learning and adaptation	AI continuously learns from new data and feedback loops, refining models to enhance the accuracy and reliability of both UDT's strategic forecasts and UB's operational actions.	Adaptive learning for real-time resource deployment and disaster response planning.
**Aspects**	**Roles and Functions of Artificial Intelligence of Things**	**Examples of Applications**
Seamless data integration	AIoT combines IoT's connectivity with AI's computational power to create a unified ecosystem where real-time data from urban sensors, IoT devices, and other sources can flow seamlessly across systems.	Centralized data collection from urban sensors for integrated dashboards.
Enabling interoperability	AIoT ensures the integration of heterogeneous CPS setups by standardizing communication protocols, aligning semantic frameworks, and supporting bidirectional data flow or exchange between UB and UDT.	Unified data frameworks for transportation and energy systems.
Real-time data processing	Through edge and fog computing, AIoT decentralizes data processing, reducing latency and improving responsiveness. This function supports UB in managing immediate urban challenges dynamically.	Dynamic energy load balancing in response to real-time demand.
Bridge between operational and strategic layers	AIoT links UB's real-time monitoring with UDT's long-term planning by ensuring that operational insights from UB inform UDT's predictive models while UDT's forecasts refine UB's real-time actions.	UB informs real-time traffic data for UDT's long-term transport infrastructure strategies.
Cross-domain optimization	AIoT supports CPSoS by enabling cross-domain collaboration and system-wide optimization, ensuring energy efficiency, transportation management, environmental monitoring, and other urban domains operate in synergy.	Integrating smart mobility, energy, and environmental initiatives in one cohesive framework.
Scalability and adaptability	AIoT provides the technological infrastructure necessary for scaling the framework to accommodate the increasing complexity of urban environments and integrating emerging technologies.	Scaling IoT networks for city expansion; integrating AI-driven robotics for urban management.
**Aspects**	**Synergy Between AI and AIoT**	**Applications**
Bridging real-time and strategic layers	AI and AIoT work concurrently to bridge the gap between real-time urban operational management (UB) and long-term strategic urban planning (UDT). While AI drives computational and analytical processes, AIoT facilitates the seamless integration of these capabilities across urban systems.	Adaptive urban ecosystems balancing real-time mobility management with predictive infrastructure planning.
Creating a cohesive ecosystem	Together, AI and AIoT enable the framework to dynamically adapt to immediate challenges (UB) while maintaining alignment with long-term goals (UDT), creating a cohesive, intelligent, and resilient urban ecosystem.	Urban systems dynamically respond to challenges such as traffic congestion and long-term environmental degradation, employing strategies that address these issues.

The interconnections among the foundational framework components and processes ensure cohesion and functionality. UB and UDT complement each other, forming a unified system where short-term operational decisions are seamlessly guided by strategic foresight. Bidirectional integration through feedback loops aligns operational actions with strategic goals, ensuring synchronization. Specifically, feedback mechanisms enable real-time data from UB to inform UDT's long-term models, while predictive insights from UDT refine UB's operational processes. This iterative feedback loop sustains continuous alignment between immediate actions and long-term objectives. The CPSoS framework's scalability and adaptability allow dynamic adjustments to urban systems, making it suitable for varying city sizes and evolving challenges.

The framework relies heavily on API integration hubs to facilitate seamless communication and interoperability between UB and UDT as CPSoS. APIs act as essential intermediaries, enabling these systems to exchange data, insights, and functionalities efficiently. As conduits, APIs allow UB to share real-time data, analytics, and operational insights with UDT, enhancing its functionalities. Conversely, APIs enable UDT to relay simulation results and predictive insights back to UB, ensuring that UB's real-time decision-making remains aligned with long-term strategic objectives.

The integration of APIs is further amplified by the incorporation of AIoT and CPSoS layers within the framework. Together, these technologies create a synergistic relationship between UB and UDT, fostering a feedback-driven ecosystem where their functions reinforce each other. This unified approach ensures that UB and UDT function cohesively, maximizing their respective strengths to address complex challenges and advance environmental sustainability in smart cities.

### 5.2 Key environmental sustainability outcomes of integrating Urban Brain and Urban Digital Twin

The integrated framework positions UB and UDT as synergistic systems that collaboratively enhance the environmental performance of sustainable smart cities. The integration of UB and UDT effectively tackles critical environmental challenges and drives progress across urban systems. The framework optimizes and harmonizes diverse environmental domains through the AIoT-enabled functionalities of UB and UDT. Table 11 provides an in-depth view of how the framework operationalizes AI and AIoT technologies to address immediate management needs, achieve long-term planning objectives, and integrate these elements into adaptive operations and strategies for environmental sustainability. These insights, except for the unique value derived from this synergy, are synthesized from a substantial body of the existing literature, particularly based on the extensive reviews conducted by Bibri [6,29], Bibri et al. [22], Bibri et al. [64,93], and Bibri and Huang [32], which examine applied AI and AIoT solutions for environmental sustainability and climate change in the context of sustainable smart cities and their operational management and strategic planning, including through UDT and UB.

**Table 11:** Key environmental sustainability outcomes of the integrated Urban Brain and Urban Digital Twin framework.

Environmental domains	Immediate and dynamic management (UB)	Strategic and predictive planning (UDT)	Integrated data-driven management and planning	Foundational framework benefits
**Transportation**	Real-time traffic monitoring, optimization of traffic signals, and congestion reduction through dynamic routing.	Scenario modeling for future transportation needs and long-term investment planning.	Combining real-time traffic management with predictive planning for sustainable transportation networks.	Reduced congestion, decreased travel times, lowered emissions, and improved public transportation systems.
**Energy management**	Real-time energy distribution optimization and monitoring to prevent overloads and ensure efficient usage.	Predicting energy demand patterns and planning for renewable energy integration.	Integrating real-time energy management with long-term renewable energy adoption strategies.	Enhanced energy efficiency, improved energy resilience, maximized renewable energy utilization, minimized energy losses, and cost savings.
**Infrastructure development**	Monitoring construction processes and detecting potential risks during real-time infrastructure operations.	Strategic modeling for future urban infrastructure demands, including sustainability standards.	Aligning dynamic infrastructure monitoring with future urban development plans.	Improved infrastructure resilience and adherence to sustainability standards.
**Air quality**	Dynamic pollution level monitoring and alerts for mitigation actions.	Forecasting pollution trends and planning long-term air quality improvement projects.	Synchronizing real-time pollution controls with strategic air quality improvement projects.	Cleaner urban environments, improved public health outcomes, and compliance with air quality regulations.
**Waste management**	Optimizing waste collection schedules and routes in real-time to reduce operational inefficiencies.	Long-term planning for waste recycling programs, facility expansions, waste-to-energy systems, and circular economy.	Harmonizing waste management processes with long-term recycling and reduction targets and resource recovery strategies.	Reduced operational costs and landfill dependency, efficient recycling processes, and sustainable waste-to-energy transitions.
**Water management**	Real-time monitoring of urban water usage and detecting leakage in water distribution systems.	Planning for water resource allocation under future urban growth scenarios and flood prevention.	Merging real-time water monitoring with long-term water conservation and urban flood management.	Enhanced water security, reduced wastage through proactive management, and mitigated flood risks.
**Biodiversity**	Detection of changes in urban green spaces and rapid restoration measures.	Predictive analysis for urban biodiversity preservation and habitat restoration.	Integrating biodiversity actions with strategic urban ecological preservation.	Improved ecosystem services, greater biodiversity preservation, and enhanced urban livability and ecological balance.
**Climate mitigation**	Monitoring real-time emissions and implementing corrective measures.	Developing carbon reduction scenarios and transition plans for clean energy adoption.	Combining real-time emission reductions with long-term decarbonization policies and predictive mitigation strategies.	Lower greenhouse gas emissions, improved climate policy alignment, and accelerated transitions to carbon neutrality.
**Climate adaptation**	Dynamic response to extreme weather events such as floods and heatwaves.	Forecasting climate risks and planning adaptive infrastructure projects.	Coordinated frameworks for disaster resilience, integrating real-time response with future-proofing strategies.	Improved resilience against climate-related risks, enhanced urban safety, and minimized disaster impacts.
**Resource optimization**	Real-time monitoring and allocation of urban resources to optimize usage.	Strategic planning for long-term optimization of resources across urban domains.	Integrating real-time resource monitoring with strategic optimization initiatives.	Optimal use of urban resources, significant reduction in operational costs, and greater adaptability to changing demands.

The outcomes presented in Table 9 underscore the significant contributions of the proposed framework to advancing sustainable smart city development. The framework encapsulates the overarching benefits of this synergy by integrating UB and UDT functionalities across diverse environmental domains. Notably, it highlights the unique advantages achieved through the fusion of immediate and dynamic management with predictive and strategic planning capabilities within a cohesive system.

This synthesis builds on prior research to address critical gaps in sustainable urban development, providing both empirical and theoretical foundations for harnessing the environmental synergies of UB and UDT. The framework adapts these concepts to specific environmental domains by extending existing theories of real-time urban management and strategic planning. Each domain is addressed through AI and AIoT techniques, supported by empirical evidence and theoretical advancements, delivering a comprehensive solution to the multifaceted challenges of environmental sustainability in smart cities.

## 6 Discussion

The integration of UB and UDT within an AIoT-driven CPSoS framework presents an innovative approach to advancing environmental goals in sustainable smart cities. This study introduced a pioneering foundational framework that synergistically integrates the real-time analytical and operational capabilities of UB with the predictive modeling and simulation capabilities of UDT. The framework addresses critical challenges of dynamic urban management and long-term urban planning by bridging these distinct yet complementary capabilities. This discussion section summarizes and interprets the findings in the context of the research objectives, compares the results with existing literature, explores their implications for research, practice, and policymaking, and addresses the challenges, limitations, and directions for future research.

### 6.1 Summary of findings and their interpretation

The study achieved its objectives, yielding significant insights into the integration of UB and UDT for enhancing the environmental performance of sustainable smart cities. Concerning the first objective, it reveals that sustainable smart cities increasingly depend on emerging technological paradigms to address multifaceted environmental challenges. AIoT emerges as a central enabler, generating real-time operational and long-term strategic insights in the domain of urban management and planning. Meanwhile, CPSoS frameworks provide the structural and functional foundation for scaling and interconnecting large-scale AIoT systems or multifaceted CPS setups, enabling cross-domain collaboration, system-wide optimization, and adaptive decision-making. However, significant gaps persist in harmonizing these technological paradigms within emerging city platforms, particularly in addressing interoperability, scalability, and holistic integration.

Regarding the second objective, UB and UDT demonstrate distinct yet complementary roles within AIoT-driven ecosystems. UB excels in real-time monitoring and advanced data analytics, delivering actionable insights to facilitate immediate decision-making and enhance operational efficiency in urban management. Conversely, UDT specializes in predictive modeling and scenario testing, supporting strategic planning and sustainability initiatives. Together, these systems demonstrate great potential to synergize, aligning short-term urban responses with long-term urban objectives and bridging real-time operations with strategic foresight.

As regards the third objective, UB and UDT significantly contribute to data-driven urban management and planning, particularly in advancing environmental sustainability and climate resilience. UB facilitates resource efficiency, energy optimization, environmental protection, and traffic and transport management. In contrast, UDT enables informed planning for resource allocation, infrastructure development, energy forecasting, renewable energy deployment, climate mitigation and resilience, biodiversity preservation, and sustainable urban design. Together, they exemplify the potential of AIoT-driven CPSoS to deliver holistic, scalable solutions for sustainable smart cities, especially in addressing environmental challenges. However, gaps remain in integrating localized and global urban dynamics, emphasizing the need for further refinement of these frameworks to fully leverage their potential in light of emerging technological convergences.

Concerning the fourth objective, the development of the proposed AIoT-driven CPSoS framework underscores the untapped potential of integrating UB's real-time analytics using stream processing capabilities with UDT's predictive analytics using simulation models capabilities. **Stream processing** is a computational approach and data management technique that involves ingesting a continuous data stream in real-time or near real-time to quickly analyze, filter, transform, or enhance the data. Unlike batch processing, which requires data to be collected, stored, and processed later, stream processing analyzes and acts on the data as soon as they are generated. Once processed, the data are passed to an application or another stream-processing engine for further use. Furthermore, the framework emphasizes a bidirectional feedback loop, where UB's real-time insights inform UDT's strategic models dynamically, while UDT's simulations refine UB's operational decisions. It provides a scalable and structured approach to integrating UB and UDT, leveraging AIoT's computational capabilities and CPS's operational efficiencies. It demonstrates a novel methodology for enhancing data-driven urban management and planning systems through unified strategies. Moreover, the inherent scalability and adaptability of CPSoS allow the framework to tackle diverse urban complexities.

### 6.2 Implications for research, practice, and policymaking

This study carries significant implications for advancing research, informing practical applications, and shaping policymaking in the dynamic landscape of sustainable smart cities.

The proposed AIoT-driven CPSoS framework contributes to the theoretical foundations of sustainable urban development by addressing the interplay between emerging city platforms for data-driven urban management and planning, thereby advancing environmental sustainability in smart cities. It lays the groundwork for a holistic approach to navigating the complexities of urban systems. This research opens new avenues for future studies to explore the scalability, adaptability, and coordination of integrated city platforms at various spatial scales and in diverse urban settings, offering insights into how ongoing technological convergences can be tailored to meet the specific demands of urban domains essential for achieving interrelated environmental goals.

The framework underscores the potential for extending AIoT capabilities to enhance the functional synergies of multifaceted CPS setups across critical areas of data-driven urban management and planning. Its emphasis on bridging operational efficiency with strategic foresight encourages interdisciplinary exploration into multi-domain interoperability, resilience, and applicability. This provides fertile ground for research spanning urban management, urban planning, environmental science, computer science, data science, control systems engineering, and systems engineering. Moreover, the study emphasizes the importance of interdisciplinary collaboration and system-level coordination in designing holistic systems for advancing environmental sustainability goals in smart cities. In doing so, the framework advances the research agenda in sustainable smart city development, presenting opportunities for innovation across both theoretical and applied dimensions.

For urban planners, engineers, and system designers, the proposed framework provides actionable strategies to enhance the operational and strategic capacities of urban systems. It equips practitioners with the tools to design adaptive and scalable urban systems. This framework serves as a blueprint for practitioners, enabling them to operationalize the synergies between UB and UDT, thereby enhancing informed decision-making, optimizing resource utilization, reducing carbon emissions, and fostering urban resilience in the face of climate and societal challenges. The practical implications of the foundational framework are particularly evident in critical urban domains. Its application can lead to tangible improvements in this regard, ensuring that urban systems are equipped to handle dynamic demands and unforeseen conditions by facilitating the creation of interconnected urban ecosystems. Furthermore, the framework promotes a holistic approach to sustainable urban development by aligning operational management with strategic planning. Practitioners can utilize integrative principles to address interconnected challenges.

The proposed framework is inherently adaptable to varying socio-economic conditions, enabling its practical application in regions outside Europe. Urban systems across different regions often face distinct challenges shaped by unique cultural, economic, and environmental factors. Its modularity and scalability allow it to accommodate these variations while maintaining its core principles. In resource-constrained regions, the framework prioritizes cost-effective and incremental strategies, focusing initially on high-impact, low-cost domains such as energy efficiency and waste management. This modular design supports gradual expansion, aligning with resource availability, and ensures feasibility without overwhelming existing systems.

Socio-cultural dynamics and governance structures also play a critical role in the framework's adaptability. Cities can align immediate interventions with culturally relevant long-term sustainability goals by integrating participatory approaches and leveraging localized data, thereby fostering community engagement and acceptance. Furthermore, phased technological integration tailored to regions' readiness levels ensures sustainable development. Initial reliance on foundational technologies can evolve into more advanced systems as cities grow technologically, maintaining alignment with strategic sustainability objectives.

For regions experiencing rapid urbanization, the framework addresses environmental challenges through real-time monitoring and predictive planning. Moreover, global collaboration fosters the exchange of best practices and insights, enabling regions at earlier stages of implementation to benefit from proven strategies. This adaptability ensures the framework's relevance across diverse urban contexts, creating a pathway for urban systems worldwide to achieve efficiency, resilience, and sustainability, regardless of their socio-economic starting points.

The proposed framework offers a solid foundation for policymakers to design evidence-based strategies that align short-term urban management actions with long-term planning strategies. Policymakers can develop and implement adaptive policies for short-term and long-term goals of environmental sustainability. Moreover, the framework highlights the critical role of policy interventions in fostering cross-sectoral collaborations, promoting investments in integrated urban infrastructure, and aligning regulatory frameworks to address key challenges, including data privacy, system interoperability, ethical governance, and community engagement. Policymakers can accelerate the deployment of innovative, sustainable smart city solutions while simultaneously building public trust and ensuring equitable, responsible, and ethical outcomes.

The integration of UB and UDT emphasizes the need for policies that support strategic investments in emerging technological paradigms. Policymakers can draw on this framework to develop comprehensive urban strategies that balance the pressing operational needs of cities with broader strategic goals, such as renewable energy deployment, carbon emission reduction, sustainable resource management, and climate resilience. In addition, the framework serves as a guide for creating policies that promote the scalability and adaptability of sustainable smart city initiatives across diverse urban contexts. Policies informed by this integrative framework can catalyze the transformation of urban systems into cohesive, resilient, and sustainable ecosystems that effectively address both present and future challenges.

### 6.3 Challenges and barriers

The integration of UB and UDT into a unified AIoT-driven CPSoS framework presents multifaceted challenges that span technical, computational, environmental, ethical, social, financial, and regulatory domains. These challenges stem from both technical complexities and the multidimensional and complex nature of urban environments, necessitating collaborative efforts among stakeholders to realize the full potential of the foundational framework for advancing environmental goals in sustainable smart cities.

A key challenge lies in achieving interoperability among diverse systems and technologies that constitute UB and UDT, particularly CPS setups. These systems rely on heterogeneous data sources, protocols, formats, and processes, complicating seamless integration and communication. The absence of standardized frameworks to harmonize these components may exacerbate inefficiencies in data exchange and coordination. Ensuring compatibility across IoT sensors and devices, AI models and algorithms, and CPS feedback and control mechanisms becomes especially challenging in large-scale urban ecosystems. Addressing this challenge requires the establishment of standardized communication protocols and flexible architectures that adapt to various urban domains while maintaining interoperability.

The real-time data generated by urban systems imposes significant demands on data processing and management capabilities. Advanced analytics powered by ML, DL, CV, NLP, and GenAI require substantial computational resources, often straining existing urban infrastructure. Scalability further complicates deployment, particularly in densely populated cities with high data volumes. Scaling involves accommodating an increasing number of sensors and data sources while ensuring that the integrated UB-UDT framework maintains performance under high computational loads. Developing scalable computational architectures and real-time algorithms is crucial for optimizing real-time data processing and predictive modeling in expanding urban environments.

While the framework aims to promote environmental sustainability, its implementation poses potential risks. Real-time analytics and large-scale simulations using advanced AI models and algorithms can demand significant computational and energy resources, potentially undermining sustainability gains (e.g., Refs. [6,55,[167], [168], [169], [170], [171]]). Energy-efficient algorithms, edge computing solutions, and sustainable computing practices are crucial for aligning technological advancements with environmental objectives (e.g., Ref. [[172], [173], [174], [175]]). Ensuring that UB and UDT operate efficiently without exceeding resource limitations is key to sustainable and responsible deployment.

The integration of UB and UDT within an AIoT-CPSoS framework raises pressing ethical and governance concerns. Key issues include ensuring data protection, mitigating algorithmic biases, and maintaining transparency in decision-making processes to foster public trust and confidence. The social implications extend to promoting fairness, gender equality, social equity, and accountability, while addressing potential disparities and digital divides in access to technology and its benefits. Aligning diverse stakeholder interests—urban planners, policymakers, technology providers, and community representatives—adds another layer of complexity. Transparent communication channels and inclusive approaches are crucial for ensuring equitable representation and inclusivity.

The interconnected nature of CPSoS increases the risk of cyber threats, such as data breaches, malicious attacks, and unauthorized access. Given that UB and UDT process sensitive data, including real-time urban operations and predictive scenarios, safeguarding data integrity and confidentiality is paramount. Robust cybersecurity measures, including advanced encryption, real-time threat detection, and resilient system architectures, are critical. These measures must also comply with data privacy regulations while maintaining public trust.

Implementing the proposed framework will require substantial financial investments in cutting-edge technologies, infrastructure upgrades, workforce development, and professional skills enhancement. These financial and capacity-building demands may create accessibility challenges for cities with limited budgets or inadequate technological resources. Ensuring equitable distribution of resources and opportunities is vital to prevent disparities in the benefits derived from the framework across urban landscapes.

The integration of UB and UDT requires interdisciplinary expertise spanning urban management, urban planning, environmental science, computer science, data science, and systems engineering. Coordinating across these fields often results in gaps in understanding and delays in implementation. Effective interdisciplinary collaboration is crucial for achieving cohesive solutions that align operational management with strategic planning. Divergent priorities and interests can impede the deployment and operationalization of the framework. Establishing clear governance structures and fostering participatory decision-making processes are critical to aligning stakeholder objectives and ensuring the framework's effectiveness.

A core challenge in integrating UB and UDT is harmonizing their distinct functionalities. Developing bidirectional feedback loops that seamlessly align short-term responses with long-term goals is crucial for advancing environmental sustainability in smart cities. This entails creating adaptive algorithms and interfaces that enable real-time analytics from UB to refine UDT's strategic simulations and vice vera, without introducing delays or inconsistencies.

The proposed framework can maximize its applicability and effectiveness while minimizing risks by systematically addressing these challenges. Collaboration among stakeholders, investments in infrastructure and human capital, and innovative approaches are essential to ensure the successful deployment of the framework. These efforts will enable sustainable smart cities to enhance their environmental performance, fostering resilient, adaptive, and efficient urban ecosystems.

### 6.4 Limitations

While the foundational framework offers a comprehensive strategy and operational model for integrating UB and UDT within a unified ecosystem, several limitations must be acknowledged to contextualize its contributions and areas for improvement.

The framework primarily emphasizes the technological, architectural, and functional aspects of AIoT-driven CPSoS, including real-time analytics, predictive modeling, computational processes, integration frameworks, and system coordination. However, it does not thoroughly address the sociocultural and sociopolitical factors that are crucial for ensuring the equitable and inclusive implementation of sustainable smart city technologies. These factors influence stakeholder engagement, policy formulation, institutional buy-in, and public acceptance, which are essential for the framework's widespread adoption and effectiveness.

A significant limitation of the framework is its reliance on the availability of advanced infrastructure, including high-speed connectivity, robust computational resources, and reliable data storage systems. In resource-constrained urban contexts, such infrastructure may be insufficient or non-existent, hindering the applicability and scalability of the framework. This poses challenges for implementing the framework in developing regions with limited technological maturity. Moreover, the accuracy and reliability of the framework are contingent on the availability of high-quality input data. Data scarcity, inconsistencies, or poor integration can significantly impact the framework's ability to deliver actionable insights, particularly in underdeveloped or resource-constrained settings. This limitation highlights the need for strategies to enhance data collection, management, and standardization in diverse urban settings. Furthermore, the framework was developed under specific assumptions about technological capabilities, urban infrastructure, and stakeholder priorities. These assumptions may not be universally applicable, particularly in cities with different levels of development, technological readiness, and governance structures. As a result, the findings and recommendations derived from this study should be interpreted with caution when applied to urban contexts that deviate from these assumptions.

While the framework prioritizes environmental sustainability as a core objective, urban stakeholders often face competing demands, such as economic development, social equity, and political considerations. Balancing these diverse priorities remains a significant challenge and requires further exploration. The framework's focus on environmental objectives may not fully align with the priorities of all stakeholders, potentially limiting its practical adoption.

Although the framework is grounded in robust theoretical and practical insights, it has not been empirically validated at scale in real-world urban scenarios. It is important to note that pilot projects and practical implementations are outside the scope of this study, which primarily seeks to establish a structured and scalable framework for other researchers and practitioners to build upon, advance, and adapt. Therefore, the framework's potential, scalability, and adaptability remain to be fully explored with practical application in real-world settings. Testing the framework in diverse urban contexts is necessary to evaluate its practical performance, refine its design, and address context-specific challenges.

Methodologically, while this study provides a comprehensive state-of-the-art review and develops an innovative foundational framework grounded in integrative analytical and synthesized insights, certain methodological limitations inherent to this approach should be acknowledged: First, the study's reliance on available literature limits its ability to capture unpublished or emerging studies that may not yet be widely documented. This could exclude innovative but less disseminated works or practices. Although care was taken to ensure an exhaustive review, the possibility of overlooking relevant contributions cannot be ruled out. In addition, the selection of academic databases, while extensive, may introduce bias in the scope and diversity of reviewed studies, potentially excluding contributions published in less accessible sources.

Third, the thematic literature review methodology, while effective for synthesizing insights across diverse domains, is inherently interpretative, potentially relying on subjective judgment to categorize themes and integrate findings. Despite a systematic approach, this process may be influenced by the scope of selected studies and their framing, introducing potential biases in how themes and trends are identified and synthesized. Lastly, the focus on literature from 2020 to 2024 ensures recency but may omit foundational studies developed before this period that remain relevant to the topic.

The study positions itself transparently by recognizing these limitations, paving the way for further refinement, validation, and investigation of its contributions. This entails addressing sociocultural, infrastructural, theoretical, practical, and methodological constraints to ensure broader relevance, applicability, and impact. These considerations also provide a roadmap for future research aimed at bridging the gaps between theory, technology, practice, methodology, and policy in sustainable smart city development.

### 6.5 Suggestions for future research

Building on the challenges and limitations highlighted in this study, several promising opportunities emerge for future research to further advance the field of sustainable smart city development. Table 12 provides a concise summary of key recommendations and potential research avenues that aim to address existing gaps and enhance the framework.

**Table 12:** Summary of suggestions for future research directions.

Focus areas	Research directions
**Empirical validation**	Future research should focus on implementing and testing the proposed framework in diverse urban settings to evaluate its practical performance and scalability. Empirical validation across varying urban contexts—ranging from highly urbanized cities to resource-constrained areas—can provide valuable insights into the framework's effectiveness, adaptability, and generalizability. Pilot projects and case studies would help refine the framework by identifying real-world constraints and solutions.
**Sociocultural and political dimensions**	Further studies are needed to investigate the sociocultural and sociopolitical factors that influence the adoption and implementation of the framework. Investigations into public trust, stakeholder engagement, participatory decision-making, equitable access to technology and infrastructure, policy alignment, and institutional support can provide a more holistic understanding of the barriers to adoption. Research in this area can also guide strategies to foster inclusivity, transparency, and accountability engagement in sustainable smart city initiatives.
**Data quality and standardization**	Given the framework's reliance on high-quality data, future research should address challenges related to data collection, integration, and standardization. Exploring innovative approaches such as decentralized data architectures, blockchain for data integrity, and advanced data fusion techniques can help overcome issues of data scarcity and inconsistencies. Comparative studies across cities with different data infrastructures could identify best practices for improving data reliability.
**Energy-efficient computing**	Future work should prioritize the development of energy-efficient algorithms and computational methods to reduce the environmental footprint of real-time analytics and large-scale simulations using advanced AI models, particularly those from the DL and GenAI subdomains. Investigations into the integration of edge computing and distributed cloud systems, as well as green computing practices within this framework, can align technological advancements with sustainability goals.
**Functional synergies of cpsos**	Research should delve deeper into enhancing the functional synergies of CPSoS by exploring advanced bidirectional feedback loops between UB and UDT. Developing adaptive algorithms and real-time interfaces to align UB's operational analytics with UDT's strategic simulations could improve the framework's responsiveness to dynamic urban challenges.
**Multidisciplinary approach**	The integration of UB and UDT within CPSoS demands interdisciplinary expertise. Future research should focus on fostering collaborations among urban planners, computer scientists, environmental researchers, system engineers, and policymakers. Cross-domain studies can provide a comprehensive understanding of the interplay of science, technology, environment, and society, thereby enhancing the framework's applicability.
**Cybersecurity and ethics**	Given the sensitive nature of urban data, future research should address advanced cybersecurity measures to safeguard against threats such as data breaches and unauthorized access. Investigating ethical frameworks for AIoT-driven urban systems, including algorithmic fairness, bias mitigation, and ethical governance, will ensure the responsible deployment of technology.
**Scalability and adaptability**	To accommodate diverse urban scales, future research should examine strategies for scaling the framework without compromising its efficiency or functionality. Investigating modular designs and adaptive frameworks capable of accommodating varying urban complexities would enhance its scalability. Studies on the adaptability of the framework to different cultural, social, economic, and environmental contexts would further refine its universal applicability.
**Integration of emerging technologies**	Research should explore the integration of emerging technologies, such as quantum computing, 6G communication networks, and GenAI, to enhance the computational capabilities and efficiency of the framework. These advancements could significantly improve real-time data processing, predictive modeling, synthetic data generation, and data augmentation capabilities, enabling more sophisticated urban management and planning solutions.
**Policy implications**	Future studies should investigate the policy implications of integrating UB and UDT into sustainable smart city governance structures. Research on stakeholder dynamics, participatory governance models, and regulatory frameworks could identify pathways to align policy interventions with technological advancements. Emphasizing collaborations among stakeholders, including government agencies, private sectors, and community organizations, will strengthen the framework's implementation.
**Competing urban priorities**	Balancing environmental sustainability with other social and economic priorities remains a key area of ongoing research. Developing methodologies to reconcile these competing priorities while maintaining stakeholder consensus could enhance the framework's acceptability and alignment with urban goals.
**Methodological improvement**	Future research should focus on expanding the scope of literature reviews to include a wider range of perspectives and emerging insights, diversifying academic databases to mitigate selection bias, and incorporating quantitative methods such as bibliometric analysis or mixed-method approaches to complement thematic analysis and reduce interpretative subjectivity. In addition, revisiting foundational studies beyond the 2020–2024 timeframe could offer valuable historical context and enrich the understanding of balanced contributions to the field of sustainable smart city development.

Addressing these research directions allows future studies to refine and expand the proposed framework, improving its relevance and effectiveness in driving sustainable smart city development. Such efforts are instrumental in bridging existing knowledge gaps and fostering the development of innovative, inclusive, and adaptable strategies to tackle the complex challenges posed by urbanization, ecological degradation, and resource scarcity.

## 7 Conclusion

This study addressed critical gaps in the rapidly burgeoning field of sustainable smart cities by introducing a pioneering foundational framework—Artificial Intelligence of Things for Sustainable Smart City Brain and Digital Twin Systems. This framework integrates the distinct yet complementary functionalities of UB and UDT as AIoT-driven CPSoS, creating a unified system that bridges real-time operational urban management and strategic predictive urban planning. In doing so, it provides a holistic and scalable approach to tackling the multifaceted and interconnected challenges of environmental sustainability in smart cities.

The study achieved its objectives by offering critical insights into the integration of UB and UDT to enhance sustainable urban development practices. It revealed the central role of AIoT and CPS in addressing urban complexities, emphasizing their significant potential for system-wide optimization, cross-domain collaboration, and adaptive decision-making for advancing environmental sustainability goals in smart cities. Moreover, UB and UDT were shown to play distinct but complementary roles within AIoT-driven ecosystems. UB demonstrated its strength in real-time urban management through its capabilities in dynamic monitoring, in-depth analytics, and immediate decision-making, enabling effective responses to urgent and evolving challenges. In contrast, UDT showcased its expertise in predictive modeling and scenario planning, leveraging advanced simulations and long-term forecasting to inform and shape sustainability strategies.

Moreover, compared to UB, UDT contributes more significantly to environmental sustainability due to its advanced functionalities, which enable the anticipation and mitigation of long-term environmental challenges. UDT supports resource allocation, energy forecasting, renewable energy deployment, climate scenario analysis, biodiversity preservation, and sustainable urban design—domains that require forward-looking strategies and data-driven planning. UDT's contributions go beyond operational improvements by addressing systemic and structural environmental challenges, offering pathways to achieve enduring sustainability goals. Together, UB and UDT bridge short-term operational needs with overarching long-term objectives, providing actionable insights across various domains of environmental sustainability. Their synergy within CPSoS frameworks exemplifies their potential to address environmental challenges holistically, integrating dynamic real-time responses with strategic interventions.

The proposed framework emphasizes a synergistic relationship between UB and UDT, where UB's real-time insights dynamically inform UDT's strategic simulations, whose resulting forecasts, in turn, refine UB's operational decisions. This bidirectional feedback loop ensures synchronized responses to immediate challenges while aligning with long-term objectives to drive environmentally sustainable smart city development. The framework establishes a structured and scalable solution for managing and planning urban systems more effectively by harnessing the computational and analytical capabilities of AIoT and the operational and adaptable coordination mechanisms of CPSoS.

Within this framework, real-time management and predictive planning are complementary approaches that represent two distinct but interconnected temporal modalities of urban intelligence. Their conceptual alignment lies in their differing functions and focal points—reactive responses in the present versus anticipatory strategies for the future. This deliberate juxtaposition of temporal modalities emphasizes their role in forming a continuous spectrum of urban decision-making. Together, they constitute a temporal continuum—responding to current dynamics while simultaneously preparing for long-term developments. Their synergistic integration underscores how they can mutually reinforce environmental outcomes in sustainable smart city systems.

This research offers key advancements to data-driven urban management and planning, particularly in promoting environmental sustainability in smart cities. It introduces a unified framework that integrates UB and UDT, bridging traditionally siloed domains of urban management and planning to better align operational actions with long-term goals. It also demonstrates how these technologies enable intelligent decision-making, real-time optimization, and strategic foresight by emphasizing the integral role of AIoT and CPS in equipping cities to dynamically respond to complex environmental challenges. Furthermore, it substantiates the synergistic potential of UB and UDT functions, amplifying urban efficiency, resilience, and environmental performance through integrated processes and practices. The proposed framework provides actionable solutions for advancing environmentally sustainable urban development by offering a structured blueprint for researchers, practitioners, and policymakers. Equipped with this framework, stakeholders are better positioned to design adaptive and collaborative urban ecosystems that harmonize real-time management with strategic planning.

The framework advances the broader goals of sustainable smart city development by unifying dynamic urban operations and strategic foresight. It provides actionable pathways for optimizing resource utilization, reducing carbon emissions, and fostering resilience, demonstrating the power of aligning operational efficiency with predictive planning. Through its holistic and integrative approach, the proposed framework positions itself as a transformative model for fostering environmentally conscious, adaptive, and efficient urban ecosystems, paving the way for an innovative urban future.

However, several limitations and challenges need to be addressed and overcome for the framework to be fully realized and widely implemented. These limitations and challenges, which were extensively discussed in the previous section, highlight the complexities inherent in achieving seamless integration and scalability across diverse urban contexts. Addressing these obstacles is crucial to unlocking the full potential of the proposed framework and ensuring its long-term viability and impact.

## CRediT authorship contribution statement

**Simon Elias Bibri:** Writing – review & editing, Writing – original draft, Visualization, Methodology, Investigation, Data curation, Conceptualization. **Jeffrey Huang:** Writing – review & editing.

## Declaration of competing interest

The author declares that they have no known competing financial interests or personal relationships that could have appeared to influence the work reported in this paper.
